# Exploratory study of an anti-PD-L1/TGF-β antibody, TQB2858, in patients with refractory or recurrent osteosarcoma and alveolar soft part sarcoma: a report from Chinese sarcoma study group (TQB2858-Ib-02)

**DOI:** 10.1186/s12885-023-11390-4

**Published:** 2023-09-15

**Authors:** Lu Xie, Xin Liang, Jie Xu, Xin Sun, Kuisheng Liu, Kunkun Sun, Yuan Li, Xiaodong Tang, Xianan Li, Xing Zhan, Xiaohui Niu, Wei Guo

**Affiliations:** 1 Musculoskeletal Tumor Center, Peking University People’s Hospital, Beijing, 100044 China; 2 Pathology Department, Peking University People’s Hospital, Beijing, 100044 China; 3 Radiology Department and Nuclear Medicine Department, Peking University People’s Hospital, Beijing, 100044 China; 4 Orthopedic Oncology Department, Hunan Cancer Hospital, Changsha, 410013 China; 5 Medical Oncology, Sun Yat-sen University Cancer Center, Guangzhou, 510060 China; 6 Orthopedic Oncology Department, Beijing Jishuitan Hospital, Beijing, 100035 China

**Keywords:** PD-L1/TGF-β, Alveolar Soft Part Sarcoma (ASPS), Osteosarcoma, Tumor immune microenvironment

## Abstract

**Background:**

Novel and effective immunotherapies are required for refractory or recurrent sarcomas. Transforming growth factor-beta (TGF-β) is a diverse regulatory and fibrogenic protein expressed in multiple sarcoma tumors that promotes epithelial-mesenchymal transition and excessive deposition of extracellular matrix. This study evaluated the efficacy and safety of the anti-PD-L1/TGF-β antibody TQB2858 in patients with refractory osteosarcoma and alveolar soft part sarcoma (ASPS).

**Methods:**

This single-arm phase 1b exploratory study included patients with refractory osteosarcoma or ASPS who had previously undergone at least two lines of systemic therapy. Patients were administered 1200 mg of TQB2858 once every 3 weeks. The primary endpoint was objective response rate (ORR), with null and alternative hypotheses of ORR ≤5% and ≥20%, respectively. Exploratory biomarker analyses using immunohistochemistry (IHC) staining (for PD-L1 and TGF-β) were performed on pre-treatment tumor samples.

**Results:**

Eleven eligible patients were included in this study. TQB2858 did not demonstrate evidence of efficacy as 0/5 osteosarcomas had any objective response, while 2/6 ASPS showed a partial response. The median progression-free survivals were 1.51 (1.38, Not Evaluable) and 2.86 (1.38, Not Evaluable) months for the osteosarcoma and ASPS groups, respectively. None of the administered cycles met the criteria for unacceptable toxicity. Other Grade 3 toxicities included abnormal liver function and elevation of γ-glutamyl transferase. IHC analysis revealed that functional enrichment in the TGF-β pathway or PD-L1 was not associated with treatment outcomes.

**Conclusions:**

The combination of PD-L1 and TQB2858 did not significantly improve the ORR in patients with recurrent osteosarcoma. However, it improved immunogenic responses in ASPS, even after progression upon anti-PD-1/PD-L1 therapy, with an acceptable safety profile. IHC profiling with pathway enrichment analysis may not have any predictive value for survival outcomes.

**Trial registration:**

Prospectively registered in the Ethical Review Committee of Peking University People’s Hospital. The trial registration number is 2021PHA105-001 and 2021PHA140-001 and the registration date was March 2, 2022.

ClinicalTrials.gov Identifier CTR20213001 and CTR20220390

**Supplementary Information:**

The online version contains supplementary material available at 10.1186/s12885-023-11390-4.

## Background

Sarcomas are a rare, heterogeneous family of mesenchymal tumors, most of which are in an immunosuppressive state, with fewer responses to immune checkpoint inhibition than other adult tumors [1]. Among these tumors, osteosarcomas are characterized by structural alterations that further limit their potential immunogenicity [2]. Therefore, clinical responses to immune checkpoint blockade (ICB) for osteosarcoma are generally disappointing [3–6]. However, alveolar soft part sarcoma (ASPS) is a rare, translocation-driven sarcoma characterized by indolent behavior but with early evidence of metastatic spread [7]. Although the intrinsic mechanism remains under investigation, it seems to be particularly sensitive to ICB, with response rates of more than 40% after receiving anti-programmed cell death-1/programmed cell death ligand-1 (PD-1/PD-L1) monotherapy [7]. Besides ICB, treatment options for ASPS usually also include various anti-angiogenesis tyrosine kinase inhibitors (TKIs) [8]. Nevertheless, as a long-term therapeutic strategy, the toxicity of TKIs is incomparable to that of immunotherapy [9, 10]. Therefore, there is an urgent need to identify novel and effective immunotherapies for these tumors.

Transforming growth factor-β (TGF-β), is a diverse regulatory and fibrogenic cytokine, which is expressed on multiple cells including mesenchymal stem cells (MSCs) and regulates cell cycle, migration, and immune response [11]. Preclinical research has shown that osteosarcoma cells educate these MSCs by secreting TGF-β-containing extracellular vesicles that drive the formation of metastatic foci within the lungs [12]. Ji et al. [13] also revealed that elevated IFN-γ signaling indicates the anti-tumor immune response in metastatic osteosarcoma thrombus by paired samples single-cell and bulk RNA sequencing data. Liu et al. [14] used a comprehensive landscape of TGF-β-related signatures to predict prognosis, immune characteristics, and therapeutic response for osteosarcoma. Meanwhile, Genin et al. demonstrated that metastatic lesions of ASPS included abundant activated stromal myofibroblasts, which exhibited TGF-β-dependent, hypoxia-regulated cytoglobin [15]. Moreover, the release of TGF-β from tumor and stromal cells also facilitated angiogenesis and immune escape to mediate tumor invasion and migration through reshaping the tumor microenvironment [11].

TQB2858 is a new bifunctional fusion protein composed of a monoclonal antibody against PD-L1 fused with the extracellular domain of TGF-β receptor. *In vitro* and preclinical studies indicated that it had a high affinity for PD-L1, TGF-β1, and TGF-β3 and exhibited high PD-L1 target occupancy. This agent also showed an acceptable safety profile in a 1A dose-escalation phase trial. In this study, we aimed to investigate the efficacy and safety of TQB2858 monotherapy in patients who had undergone at least two lines of systemic therapy for pathological types, mainly osteosarcoma and ASPS. The primary endpoint was the objective response rate (ORR) at 12 weeks, which is commonly used in prospective studies of patients receiving immunotherapy. In addition, we performed a biomarker analysis based on immunohistochemistry (IHC) to assess whether these tests had a predictive value for the outcomes of this agent. We aimed to provide viable and valuable options for patients with advanced osteosarcoma and ASPS with IHC correlates.

## Methods

### Study design and participants

In this nonrandomized, multicenter, phase IB study (dose-expansion part), we included adult patients (age ≥ 18 years) with histologically proven advanced osteosarcoma (having undergone at least two lines of systemic therapy) or ASPS; an Eastern Cooperative Oncology Group performance status [16] of 0 to 2; adequate renal, liver, and bone marrow function [hemoglobin ≥ 8.0 g/dL; absolute neutrophil count ≥ 1,500 cells/mL; platelet count ≥ 90,000 cells/mL; bilirubin concentration ≤ 1.5X upper limit of normal (ULN); aspartate aminotransferase (AST) and alanine aminotransferase (ALT) concentrations ≤ 2.5X ULN or ≤ 5X ULN in patients with liver metastasis, respectively, and calculated creatinine clearance ≥ 60 mL/min]. Patients who had received fewer than two courses of treatment and those with uncontrolled active central nervous system metastasis and/or carcinomatous meningitis, known hypersensitivity to administered drugs, or preexisting adverse events of grade 2 or higher (as assessed using the NCI Common Terminology Criteria for Adverse Events version 5.0) were excluded. This study was approved by the ethics committees and institutional review boards of the participating institutions (Peking University People’s Hospital and Hunan Cancer Hospital) and conducted in accordance with Good Clinical Practice and the Declaration of Helsinki. Written informed consent was obtained from all patients. All authors had access to the study data, and reviewed and approved the final manuscript.

### Procedures

All patients received TQB2858 (1800 mg) once every 3 weeks, which was delivered as a 20-min to 1-h intravenous infusion. Doses were reduced or delayed in subsequent cycles for certain patients, depending on toxicity. Intrapatient dose escalation was not permitted. Treatment was continued until any of the following occurred: disease progression, an intercurrent illness that prevented additional treatment, a treatment delay of greater than 3 weeks for any reason, unacceptable adverse event(s), general or specific changes in the patient’s condition that rendered additional treatment unacceptable (as judged by the investigator), or withdrawal of patient consent.

### Tumor sample collection and immunohistochemical staining

Tumor samples were collected before the initiation of treatment using formalin-fixed paraffin-embedded (FFPE) tissues at the patient’s discretion. If the tumor content of the sample was estimated to be ≥40% on pathologic examination, IHC was used to detect the expression of PD-L1 and TGF-β1 in FFPE sections. (TGF-β; clonePD00-17, Cat#HA721143; HVABIO; Hangzhou, China); (PD-L1; clone 22C3, Cat#A1645; ABclonal; Wuhan, China). PD-L1 tumor expression was determined by IHC performed at a central laboratory (22C3clone, Cell Signaling Technology, Shanghai, China) and calculated as a combined positive score (TPS, defined as the number of PD-L1 staining tumor cells out of the total number of tumor cells, multiplied by 100). TGF-β1 was centrally detected by IHC (PD00-17 clone, Cell Signaling Technology) and presented as histochemical score (H-score, defined and calculated as the product of the intensity score and proportion) in extracellular matrix and immune cells. Immunostaining results were interpreted by two senior pathologists (SKK and SDY). We also used Image-Pro Plus (version 6.0; Media Cybernetics, Inc., Rockville, MD, USA) to analyze the results.

### Outcomes

The primary endpoint of the clinical expansion part was the ORR assessed using RECIST v1.1, defined as the percentage of patients whose best overall response was confirmed as complete response (CR) or partial response (PR) [17]. The secondary endpoints included disease control rate (DCR), clinical benefit rate (CBR, defined as CR, PR, or stable disease lasting at least 24 weeks), duration of response (DoR), progression-free survival (PFS) per RECIST v1.1, and overall survival (OS). Tumor response was assessed by investigators according to RECIST v1.1 and modified RECIST 1.1, for immune-based therapeutics (iRECIST), every 6 weeks after the first administration [18]. CR or PR was confirmed with subsequent assessments after at least 6 weeks. Adverse events were evaluated for 90 days after the last dose and graded according to the National Cancer Institute Common Terminology Criteria for Adverse Events v5.0. Physical examinations, laboratory tests, and toxicity assessments were performed at baseline and prior to each treatment.

### Statistical analysis

The efficacy and safety were analyzed in all patients who received at least one dose of the study treatment. Categorical and continuous variables were compared using Fisher’s exact test and the Wilcoxon rank–sum test, respectively. PFS and OS were defined as the time from treatment initiation until evidence of disease progression or death from any cause. Survival was plotted using Kaplan–Meier curves and compared using the log–rank test. The censoring date is April 25, 2023. Statistical analyses were performed using SAS 9.4 and GraphPad Prism version 6 (GraphPad Software).

## Results

### Patient characteristics

The TQB2858-Ib-02 trial enrolled 20 patients at two centers in China between December 2021 and April 2023. Nine patients were ineligible, leaving 11 eligible patients. The baseline patient characteristics are listed in Table 1. More than half of patients had an Eastern Cooperative Oncology Group (ECOG) performance status of 0 (*n* = 6, 54.5%), others had an ECOG score of 1 (*n* = 5, 45.5%). The median age was 34.0 years (Q1, Q3, 31.0, 49.5), and 72.7% (8/11) were male. Among the 11 patients, five were diagnosed with osteosarcoma (45.5%), while the others were diagnosed with ASPS (6/11, 54.5%). All patients demonstrated lung metastasis (11/11, 100%), and three patients had metastasis to other sites (including the bone and liver). Six patients (54.5%) had received at least two prior lines of treatment. Nine patients had previous surgery (81.8%) and 1 (9.1%) had previous radiotherapy.

**Table 1:** Patient demographic and baseline characteristics

**Patient characteristics**	***N*** ** = 11**
Age, years	
Median (IQR)	34.0 (31.0, 49.5)
Gender, n (%)	
Male	8 (72.7)
Female	3 (27.3)
ECOG performance status score, n (%)	
0	6 (54.5)
1	5 (45.5)
Pathological subtypes, n (%)	
Osteosarcoma	5 (45.5)
Alveolar soft part sarcoma	6 (54.5)
Previous surgery, n (%)	
Yes	9 (81.8)
Previous radiotherapy, n (%)	
Yes	1 (9.1)
Previous systemic treatment, n (%)	
Chemotherapy	4 (36.4)
Targeted therapy	10 (90.9)
Immunotherapy	2 (18.2)
Metastatic sites, n (%)	
Lung	11 (100)
Bone	3 (27.3)
Liver	1 (9.1)
Best overall response, n (%)	
PR	2 (18.2)
SD	3 (27.3)
PD	6 (54.5)
PFS (Mean, 95% CI) months	
Osteosarcoma	1.51 (1.38, NE)
Alveolar soft part sarcoma	2.86 (1.38, NE)
Total	1.58 (1.38, 6.93)

*IQR* Interquartile range, *ECOG* Eastern Cooperative Oncology Group, *PR* Partial response, *SD* Stable disease, *PD* Progressive disease, *PFS* Progression free survival, *CI* Confidence interval, *NE* Not estimate

### Clinical outcome

**Fig. 1:**
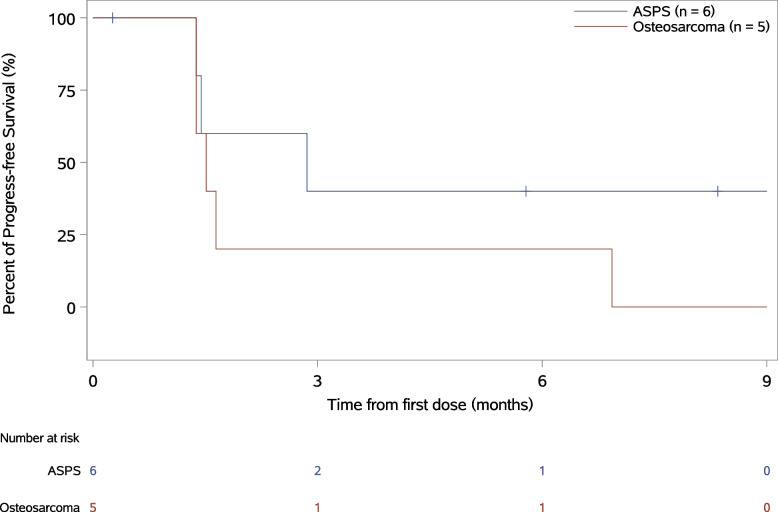
Kaplan–Meier curves for PFS. ASPS, alveolar soft part sarcoma. PFS, progression free survival

The median follow-up duration for PFS was 8.34 months (Q1, Q3, 5.78, 8.34) (Fig. 1) and for OS was 9.63 months (Q1, Q3, 7.92, 10.22). The median number of treatment cycles was 6.4 (range, 1–14). The data cut-off date was May 1, 2023. PFS events occurred in 8 patients (72.7%). In patients with osteosarcoma, the median PFS was 1.51 months (95% CI 1.38 to not evaluable (NE)). In patients with ASPS, the median PFS was 2.86 months (95% CI 1.38 to NE). The median OS was not reached in patients with ASPS or osteosarcoma (95% CI, NE, NE) (Table 1).

**Fig. 2:**
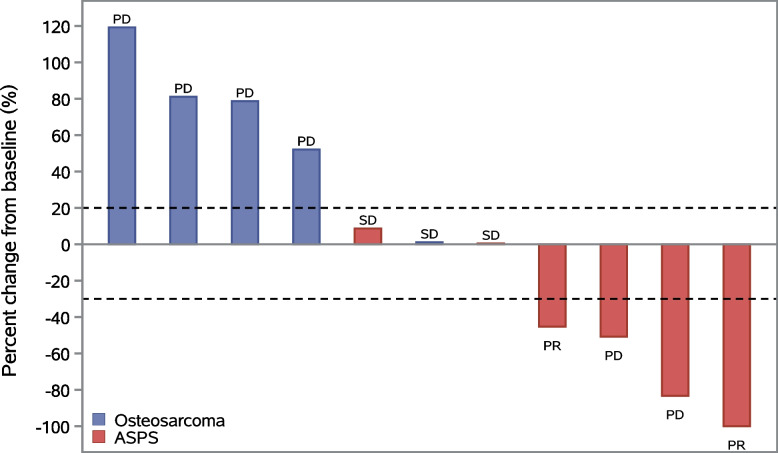
Waterfall plot for best percentage change in target lesion size in FAS. ASPS, alveolar soft part sarcoma. PD, progressive disease; SD, stable disease; PR, partial response; FAS, full analysis set

**Fig. 3:**
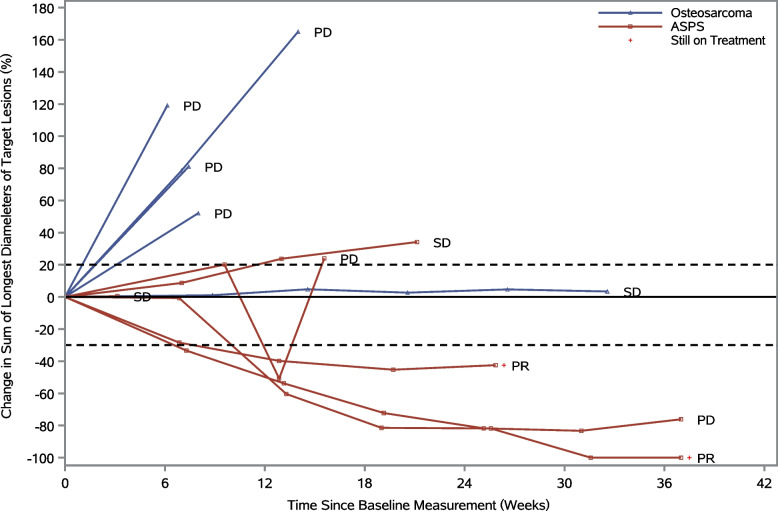
Spider plot for best percentage change in target lesion size in FAS. ASPS, alveolar soft part sarcoma. PD, progressive disease; SD, stable disease; PR, partial response; FAS, full analysis set

No patient achieved CR. Notably, 2 patients with ASPS achieved PR; the ORR and DCR of ASPS were 33.3% (2/6) and 66.7% (4/6), respectively. Tumor response was observed in two patients, and the ORR was 18.2% (2/11) for the overall population. In addition, 3 patients had stable disease (SD) (2 with ASPS and 1 with osteosarcoma), and the DCR was 45.5% (5/11) for the entire population (Figs. 2 and 3).

### Durable response for ASPS

**Fig. 4:**
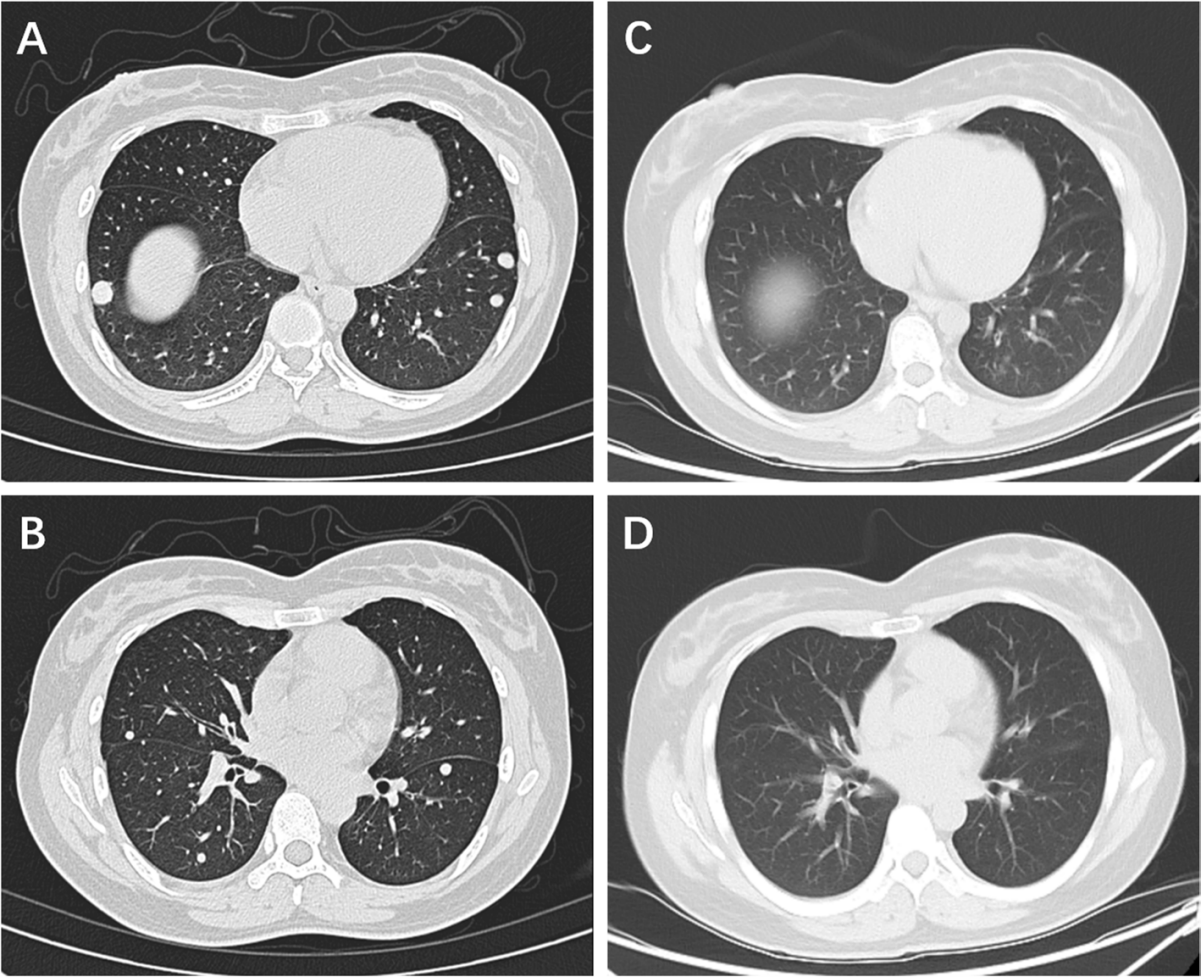
Radiological changes for the patient (PR) with metastatic alveolar soft part sarcoma. **A**, **B** Before treatment computerized tomography (CT) scans of target lesions in the lung site. **C**, **D** After treatment CT scans of target lesions in the lung site

**Fig. 5:**
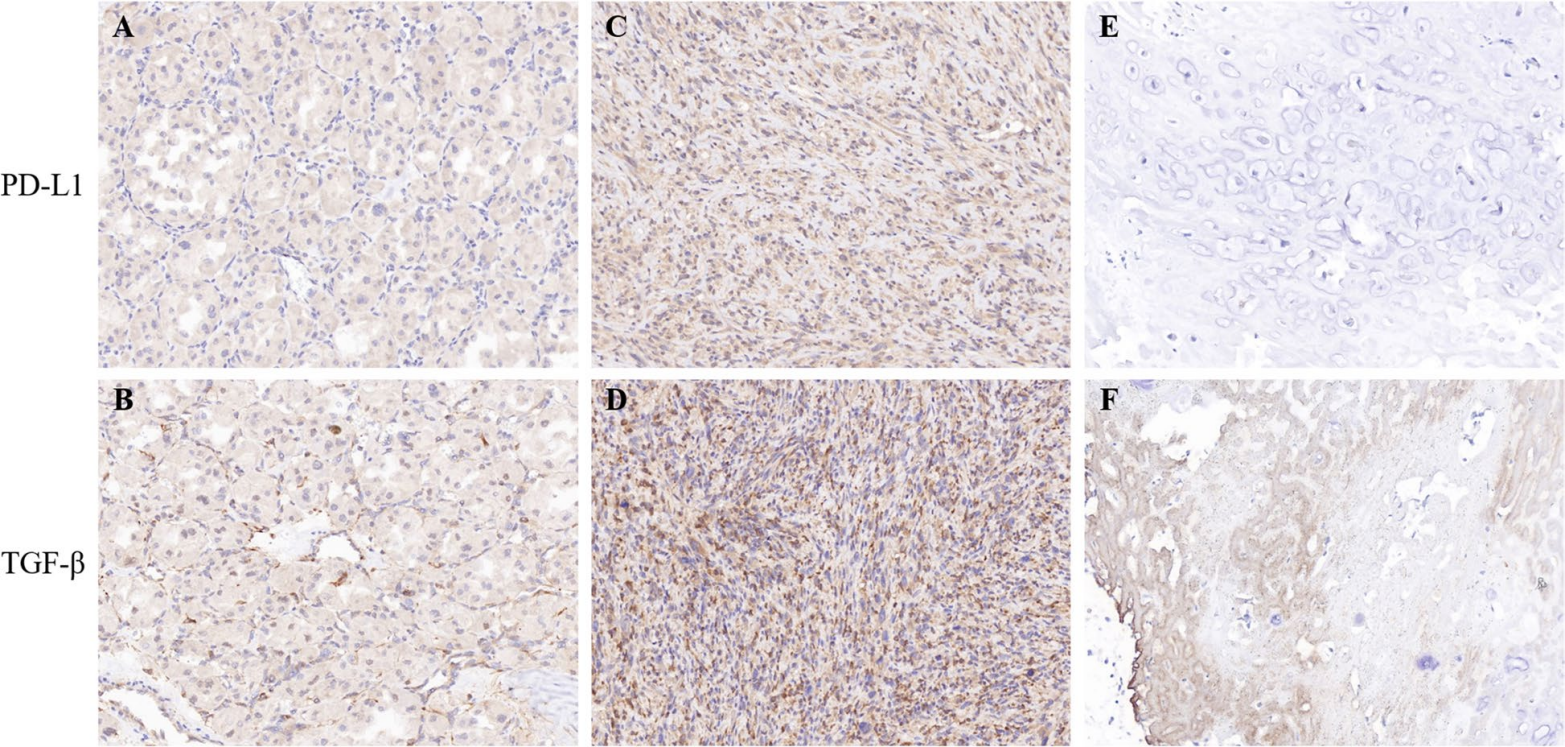
IHC staining of tumor biospecimens. **A**, **B** IHC staining of PD-L1 and TGF-β in patient 01007. **C**,** D** IHC staining of PD-L1 and TGF-β in patient 01010. **E**,** F** IHC staining of PD-L1 and TGF-β in patient 01002. IHC, immumohistochemical staining. PD-L1, programmed cell death ligand 1; TGF-β, transforming growth factor-beta

We observed two objective responses in the ASPS group with durable therapeutic effects. A 33-year-old woman presented with a left thigh mass in December 2019. The patient underwent a wide resection of the local lesion and was pathologically diagnosed with ASPS. Chest computed tomography (CT) revealed multiple metastases (more than 10 lesions with a maximum diameter of > 4 cm) in May 2021. She then received triprimab (anti-PD-1 antibody) 240 mg intravenous fusion once every 3 weeks and showed disease progression in January 2022, with the best clinical evaluation of SD. She was then administered a combination of anlotinib (an anti-angiogenesis TKIs domestically made in China) and tislelizumab (another anti-PD-1 antibody) 200 mg once every 3 weeks beginning January 5, 2022. However, in June 2022, chest CT revealed slow progression of two tiny lesions in her lungs, while she developed perforation of the appendix and abscess formation. After conservative treatment of the appendiceal abscess, she was enrolled on July 13, 2022, and received TQB2858 on July 14, 2022. After four cycles of TQB2858 infusion, her chest CT showed PR (Fig. 4). As of May 2023, her target lesions both disappeared with CR, while some residual non-target lesions could still be observed (Fig. 4). IHC analysis of FFPE tissue sections for PD-L1 and TGF-β was performed using an anti-PD-L1 antibody (clone 22C3, Cat# M3653; DAKO; Glostrup, Denmark) and the Dako Autostainer Link 48 platform, using a sample obtained from the definitive surgery of the left thigh mass. However to our surprise, we found that the specimens tested as negative no matter in PD-L1 or in TGF-β expression (Fig. 5E, F). The major adverse events were epistaxis, immune dermatitis, and low back pain. The most severe adverse event she experienced was dermatitis bullosa perineum (grade 2).

The second patient was a 31-year-old male worker who presented with a sternal manubrium mass and tenderness. Chest CT revealed a necrotic manubrium sternum with the formation of a soft tissue mass. More importantly, multiple pulmonary metastases were found. Positron emission tomography-computed tomography also demonstrated liver metastases. Subsequent puncture histopathology with hematoxylin and eosin and IHC staining confirmed the diagnosis of ASPS. He did not receive any operative or radioactive treatment before starting oral traditional Chinese medicine three times daily from August 2021. Without any evaluation of the tumor response, the treatment was stopped in May 2022. In June 2022, thoracolumbar enhanced magnetic resonance imaging (MRI) indicated bone metastasis to the sacrum and L4 and T10 vertebrae. He was then switched to anlotinib 120 mg once every 3 weeks from June 24, 2022, for three cycles. However, re-examination with enhanced chest CT and thoracolumbar MRI in August 2022 revealed multiple metastases to the bones, lungs, liver, and subcutaneous soft tissue of the right hip. He was then enrolled on September 15, 2022, and received TQB2858 on September 19, 2022. After three cycles infusion of TQB2858, enhanced CT revealed PR. As of April 2023, the patient’s target lesions were evaluated as a PR. His major adverse events were elevation of alanine aminotransferase, aspartate aminotransferase, hemobilirubin, blood lactate dehydrogenase, and D-dimer levels. Other adverse events included hypertriglyceridemia, gingival bleeding, xerostomia, constipation, and hip pain. The most severe adverse event was hip pain (grade 3).

### Safety and tolerability

All the patients experienced treatment-related adverse events (TRAE) (Table 2). The most common TRAEs of any grade were immune-mediated dermatitis (36.4%), elevated aspartate aminotransferase (AST) (27.3%), pruritus (27.3%), elevated alanine aminotransferase (ALT) (18.2%), weight loss (18.2%), and gingival bleeding (18.2%). Grade 3 or 4 TRAEs were reported in only one (9.1%) patient with abnormal liver function (elevation of γ-transglutaminase, ALT, and AST) and pruritus. TRAEs also led to treatment interruption in this patient; however, none of the patients permanently discontinued treatment because of TRAEs. In addition, serious adverse events (SAEs) occurred in two (18.2%) patients, of whom one (9.1%) had a treatment-related SAE (TRSAE). No treatment-related death occurred during the study period.

**Table 2:** Demographics of AEs with over 10% incidence

**Toxicity**	**Overall AEs**		**Grade ≥ 3 AEs**	
	**n**	**%**	**n**	**%**
Immune mediated dermatitis	4	36.4	0	0
Elevation of Aspartate aminotransferase (AST)	3	27.3	0	0
Pruritus	3	27.3	0	0
Elevation of Alanine aminotransferase (ALT)	2	18.2	0	0
Weight loss	2	18.2	0	0
Gingival bleeding	2	18.2	0	0
Elevation of N-terminal brain natriuretic peptide	1	9.1	0	0
Elevation of γ-glutamyltransferase	1	9.1	1	9.1
Constipation	1	9.1	0	0
Dermatitis bullosa	1	9.1	0	0
Acne-like dermatitis	1	9.1	0	0
Elevation of white blood cell (WBC) count	1	9.1	0	0
Iron-deficiency anemia	1	9.1	0	0
Abnormal liver function	1	9.1	1	9.1
Diarrhea	1	9.1	0	0
Autoimmune dermatitis	1	9.1	0	0
Proteinuria	1	9.1	0	0
Elevation of blood lactate dehydrogenase (LDH)	1	9.1	0	0
Elevation of blood thyroid stimulating hormone (TSH)	1	9.1	0	0
Elevation of blood platelet (PLT) count	1	9.1	0	0
Elevation of blood alkaline phosphatase (ALP)	1	9.1	0	0
Elevation of blood bilirubin	1	9.1	0	0
Hypertriglyceridemia	1	9.1	0	0

*AEs* Adverse effects

### IHC-based exploratory biomarker analysis

The tumor biospecimens of three patients were available for assessing PD-L1 and TGF-β expression (Fig. 5 A**-**F), which were from one osteosarcoma with rapid tumor progression after TQB2858 treatment (patient number 01010 in Table 3), one osteosarcoma with durable SD (patient number 01002 in Table 3), and one ASPS with a durable response who had previously progressed upon anti-PD-1 therapy (patient number 01007 in Table 3). Surprisingly, those who seemed to benefit from this compound (patient number 01002 and 01007) did not have positive PD-L1 staining, whilie 01007 seemed to have relatively positive TGF-β staining but finally progressed with new solitary subcutaneous metastasis after 7 months’ treatment. As for the initially progressed case of 01010, both PD-L1 and TGF-β seemed to be positive with strong staining (Fig. 5C, D), which urged us to further investigate the mechanism.

**Table 3:** Detailed information and treatment courses for each patient

**Patient No.**	**Pathological diagnosis**	**Previous systemic treatment**	**Previous surgical treatment**	**Duration of using current treatment, months**	**Cycles of current treatment**	**Best overall response**	**Progression free survival (PFS), days**	**Status for last follow up**
01002	Osteosarcoma	Chemo	Yes	232	12	SD	211	Alive
01003	Osteosarcoma	Radio+Chemo+Apatinib	Yes	85	5	PD	46	Alive
01005	Osteosarcoma	Chemo+Anlotinib	Yes	43	3	PD	50	Alive
01007	Alveolar soft part sarcoma	Anlotinib+Tislelizumab+Triprimab	Yes	274	14	PR	254	Ongoing
01009	Osteosarcoma	Chemo+Apatinib	Yes	43	3	PD	42	Alive
01010	Osteosarcoma	Anlotinib	Yes	22	2	PD	42	Alive
01003	Alveolar soft part sarcoma	Anlotinib	No	108	6	SD	87	Alive
06001	Alveolar soft part sarcoma	Anlotinib	Yes	1	1	SD	8	Lost follow up
16001	Alveolar soft part sarcoma	Chemo+Anlotinib	Yes	67	4	PD	44	Alive
16002	Alveolar soft part sarcoma	CPM+Anlotinib	Yes	209	10	PD	42	Alive
16003	Alveolar soft part sarcoma	TCM+Anlotinib	No	198	10	PR	176	Ongoing

*Chemo* Chemotherapy, *Radio* Radiotherapy, *CPM* Chinese patent medicine, *TCM* Traditional Chinese medicine, *PR* Partial response, *SD* Stable disease, *PD* Progressive disease

## Discussion

Although in this study, the ORR of TQB2858 did not meet prespecified efficacy criteria of more than 20% for osteosarcoma or more than 40% for ASPS, there are still several key takeaways. First, there remains a tremendous need for novel therapies, especially for immunotherapy in patients with recurrent osteosarcoma and ASPS, as demonstrated by the faster-than-expected accrual in our study, even with concurrent trials for the same patient population. Second, our current Phase 1b trial design using a benchmark approach remains a highly effective and efficient mechanism to discern the signal of the activity of novel agents in patients. Using this approach, we confirmed that the efficacy of anti-PD-L1/TGF-β is promising for inducing more immunogenic responses in ASPS. More than 45% of patients showed clinical benefits at 12 weeks. The safety profile of TQB2858 was consistent with those reported in previous studies. No unexpected safety signs or treatment-related deaths were observed. To the best of our knowledge, this is the first study to demonstrate the activity and safety toward PD-L1 and TGF-β dual antibody therapy for osteosarcoma and ASPS.

ASPS, accounting for less than 1% of all soft tissue sarcomas and occurring preferentially in young adults, is characterized by the t(X;17)(p11;q25) translocation, which codes for a chimeric ASPSCR1-TFE3 transcription factor, and is known for its sensitivity to TKI therapy with a response rate of approximately 30% [19]. Usually, translocation-related sarcomas are less immunogenic because of the poor immunogenicity of the fusion proteins and a lower mutational burden [7]. However, for ASPS, an impressive benefit from ICB was achieved, with a global ORR of 48.8% for anti-PD-1 monotherapy. The reason for this sensitivity remains unclear in histotypes characterized by poor immune infiltrates. Data regarding the tumor microenvironment of ASPS are scarce because of its rarity [20]. It is poorly infiltrated, with low CD3+, CD8+, and FoxP3+ infiltrates [1]. Thus, further laboratory research investigating the tumor microenvironment of ASPS is urgently required. Our study showed that patients who progress upon anti-PD-1 monotherapy should still acquire durable responses from the dual targeting of TGF-β and PD-L1, which should be further explored in clinical trials. However, this expansion trial has to be terminated at this time because of the too-tardy enrollment process and pharmaceutical commercial aspects. Other immunotherapy trials for soft tissue sarcomas have shown that the co-expression of TGF-β receptor II may improve the efficacy of adoptive T cell therapy-transduced T cells and overcome the inhibitory tumor microenvironment [21]. We will further investigate this mechanism in future studies.

One case in the osteosarcoma cohort (1/5, 20%) had SD lasting for almost 7 months and then progressed with one new subcutaneous lesion. Osteosarcomas, which are classified as “complex genomics,” are the most frequent form of primary bone tumors and mainly affect children, adolescents, and young adults, and determining the signaling pathways that might be targeted by specific therapies is extremely difficult [22]. Thus, a hypothesis has emerged that the particular microenvironment of these tumors may interfere with tumor cells that promote chemoresistance and the dissemination of metastasis [23]. The stroma is composed of a large number of cell types (immune cells, endothelial cells, mesenchymal stromal cells, etc.) which secrete TGF-β, a cytokine that favors the development of primary tumors and dissemination of metastases by constituting a permissive niche at primary and distant sites [12]. Abundant basic research has verified that TGF-β can thus exert its pro-tumorigenic function in primary bone tumors by promoting angiogenesis, bone remodeling, cell migration, and inhibiting immunosurveillance [24–26], but no clinical trial has proved that TGF-β determines resistance in primary anti-PD-1 therapy. Despite the unsatisfactory outcome, this study might be an initial exploration for the combination of anti-PD-1 and TGF-β. The safety profile in this study was favorable and consistent with that in previous reports [27, 28]. In this study, the rate of grade 3 or higher AEs was 27.3% (3/11), and only one patient (9.1%) had grade 3 or higher TRAEs. Meanwhile, TRSAE also occurred only in this patient, indicating that the safety of this treatment is controllable, and thus, combination therapy might be a future direction for better outcomes.

TGF-β is a diverse regulatory and fibrogenic protein with three isoforms: TGF-β1 (the most common; we performed IHC for this isoform), TGF-β2, and TGF-β3 [11]. As an inducer of cytostasis, protection and apoptosis, TGF-β initially acts to inhibit tumorigenesis [29], but later in the presence of oncogenic events and epigenetic perturbations can act as a tumor promotor [30]. TGF-β1 is found to be secreted by almost all cells, including most immune cells. TGF-β2 is mainly expressed in epithelial cells and nerve cells, while TGF-β3 is mainly expressed in mesenchymal cells. Among them, TGF-β1 is the most expressed isoform and is highly associated with the occurrence and development of many diseases. TGF-β is secreted in a precursor form binding to a propeptide, and is further cleaved by furin-type enzymes in the Golgi apparatus, transported to the extracellular matrix (ECM) in association with a latency-associated peptide (LAP), and activated in the presence of diverse molecules such as thrombospondin-1, integrins, matrix metalloproteinases (MMPs), bone morphogenetic 1 (BMP-1), and reactive oxygen species (ROS) [17]. We also aimed to identify biomarkers to identify patients who would benefit the most from TQB2858. Our IHC did not verify any relationship between the expression of PD-L1 or TGF-β and the treatment outcome, which was within expectations especially for ASPS, as no basic research has confirmed the mechanism of its immune responses. However, owing to the exploratory nature and small sample size, these preliminary findings should be interpreted cautiously and warrant further investigation.

This study had some limitations. First, and most importantly, it was a typical early phase trial with a small sample size. These two clinical-expansion cohorts need larger sample sizes with longer follow-up times to obtain a clear conclusion regarding the efficacy of this dual antibody, as well as future directions. Second, because of the rarity of ASPS, we had insufficient numbers to expand our observations, such as overcoming secondary resistance to anti-PD-1 monotherapy, which could have reduced the statistical power. By the way, patients included in the trial did not all get the same anti PD-L1 antibody before enrolled into this trial, which made the deduction mot so well-grounded. More observation with larger sample size should be obtained for the conclusion. Third, the lack of a control arm made it difficult to contextualize the findings in these two cohorts relative to the historical comparator. In addition, we performed IHC of the FFPE only for TGF-β1, which was mostly expressed in extracellular matrix other than the tumor cell membrane and made the manual interpretation difficult. Studies have reported the use of phosphorylation of SMAD2 or SMAD3, which are downstream transcription factors critical in the TGF-β pathway, to demonstrate the activation of this pathway. The effects of TQB2858 on TGF-β2 and TGF-β3 trapping require further investigation.

## Conclusions

Dual targeting of TGF-β and PD-L1 (TQB2858) did not improve outcomes in refractory or recurrent osteosarcoma and ASPS. However, it showed an acceptable safety profile, encouraging the use of combination therapy in advanced cases. Sometimes, it could overcome secondary resistance to anti-PD-1 monotherapy in selected ASPS. The PD-L1 expression and TGF-β level of mesenchymal cells might not contribute in better patient selection, which needs future validation.

### Supplementary Information

**Additional file 1: Appendix Table 1.**The relevance of TGF-beta and sarcoma subtypes in recent 6 years.

**Additional file 2: Table 1. **List of PD-L1/TGF-β dual antibody research and development. **Table 2.** summary of serum pharmacokinetic parameters after intravenous infusion of 1, 10 and 60 mg/kg TQB2858 in cynomolgus monkeys (n = 6). Table 3. Detection of anti-drug antibodies in cynomolgus monkeys after intravenous administration of TQB2858 Neutralizing activity detection rate. **Figure 1.** Mean plasma concentration-time curves of each group after intravenous injection of different doses of TQB2858 injection in cynomolgus monkeys. **Table 4.** Toxicokinetic parameters in 4-week repeated dose intravenous toxicity study in cynomolgus monkeys. **Figure 2.** Mean concentration-time curve of TQB2858 in serum of cynomolgus monkeys after intravenous injection of TQB2858. **Table 5.** List of specific laboratory test items. **Table 6.** Unlisted in NCI-CTCAE v5.0 Criteria for Judging the Severity of Adverse Events. **Table 7.** Form for Judging the Relationship between Adverse Event and Drug. 

## Data Availability

The data that support the findings of this study are available from the patient databases of Peking University People’s Hospital and Hunan Tumor Hospital separately, but restrictions apply to the availability of these data, which were used under license for the current study and so are not publicly available. However, data are available from the corresponding author upon reasonable request and with permission from the Center for Drug Evaluation of China (CDE) following the requisite protocols.

## Abbreviations

ALT: Alanine aminotransferase
ASPS: Alveolar soft part sarcoma
CBR: Clinical benefit rate
CR: Complete response
CT: Computed tomography
DCR: Disease control rate
DoR: Duration of response
FFPE: Formalin-fixed paraffin-embedded
ICB: Immune checkpoint blockade
IHC: Immunohistochemistry
MRI: Magnetic resonance imaging
MSCs: Mesenchymal stem cells
NE: Not evaluable
ORR: Objective response rate
OS: Overall survival
PD-1/PD-L1: Anti-programmed cell death-1/programmed cell death ligand-1
PFS: Progression-free survival
PR: Partial response
SAE: Serious adverse events
SD: Stable disease
TGF-β: Transforming growth factor-β
TKIs: Tyrosine kinase inhibitors
TRAE: Treatment-related adverse events
TRSAE: Treatment-related serious adverse events
ULN: Upper limit of normal
