# Supplementary material for: Exploratory study of an anti-PD-L1/TGF-β antibody, TQB2858, in patients with refractory or recurrent osteosarcoma and alveolar soft part sarcoma: a report from Chinese sarcoma study group (TQB2858-Ib-02)
Source: BMC Cancer. 2023 Sep 15;23:868. doi: 10.1186/s12885-023-11390-4 (PMC10503089; doi:10.1186/s12885-023-11390-4)
Supplement: Supplementary file 1 — Additional file 1: Appendix Table 1.The relevance of TGF-beta and sarcoma subtypes in recent 6 years. [file 12885_2023_11390_MOESM1_ESM.docx]

**Appendix Table 1** The relevance of TGF-beta and sarcoma subtypes in recent 6 years

| Sarcoma Subtypes | TGF-beta positive rate IHC (N) | Pathway Study (N) | Trials on-going |
| --- | --- | --- | --- |
| Osteosarcoma | 6%-45% (3) | TGF-β is associated with poor prognosis and promotes osteosarcoma progression via PI3K/Akt pathway activation (35) | 2 |
| ASPS | NA | None | 0 |
| Neuroblastoma | 58% (1) | None | 1 |
| Chondrosarcoma | NA | TGF-β signaling and PEG10 are mutually exclusive and inhibitory in chondrosarcoma cells (4) | 0 |
| Ewing sarcoma | 36% (1) | MicroRNA-20b promotes cell proliferation via targeting of TGF-β receptor II and upregulates MYC expression in Ewing's sarcoma cells; Wnt/β-catenin-activated Ewing sarcoma cells promote the angiogenic switch (2) | 0 |
| Desmoid Tumors | 35-60% (2) | TGF-β1 exerted its role via the canonical Smad pathway with the phosphorylation of Smad3 being crucial for TGF-β1 dependent DF cell growth and myofibroblastic differentiation (2) | 0 |
| Inflammatory sarcomas | NA | VGLL3 activates inflammatory responses by inducing interleukin-1α secretion while VGLL3 is a target of transforming growth factor β (TGF-β) signaling (2) | 0 |
| Leiomyosarcoma | NA | Overexpression of carbonyl reductase 1 inhibits malignant behaviors and epithelial mesenchymal transition by suppressing TGF-β signaling in uterine leiomyosarcoma cells (1) | 1 |
| Angiosarcoma | NA | Inhibition of Endoglin Exerts Antitumor Effects through the Regulation of Non-Smad TGF-β Signaling in Angiosarcoma (1) | 0 |
| Myxoinflammatory fibroblastic sarcoma | NA | Myxoinflammatory fibroblastic sarcoma (MIFS) has recurrent genetic features in the form of a translocation t(1;10)(p22-31;q24-25), BRAF gene fusions, and/or an amplicon in 3p11-12 including the VGLL3 gene (1) | 0 |
| Fibrosarcoma | NA | TGF-βR inhibitor SB431542 restores immune suppression induced by regulatory B-T cell axis and decreases tumour burden in murine fibrosarcoma (1) | 0 |
| Neurofibromatosis/ MPNSTs | NA | NF2 sustained TGFβ receptor 2 (TβR2) expression and reduction or loss of NF2 activated non-canonical TGFβ signaling, which reduced Raf kinase inhibitor protein (RKIP) expression via TβR1 kinase activity (2) | 0 |
| Rhabdomyosarcoma | 64% (1) | Bioinformatic analyses, performed to assess the role of 41 cytokines after RT exposure and their network interactions, suggested TGF-β, MIF, CCL2, CXCL5, CXCL8 and CXCL12 as master regulators of cancer immune escape in RMS tumors (1) | 0 |

IHC: Immunohistochemistry; N: Numbers of similar study; ASPS: alveolar soft part sarcoma; MPNSTs: Malignant peripheral nerve sheath tumors; NA: Not available.
