# Supplementary material for: Exploratory study of an anti-PD-L1/TGF-β antibody, TQB2858, in patients with refractory or recurrent osteosarcoma and alveolar soft part sarcoma: a report from Chinese sarcoma study group (TQB2858-Ib-02)
Source: BMC Cancer. 2023 Sep 15;23:868. doi: 10.1186/s12885-023-11390-4 (PMC10503089; doi:10.1186/s12885-023-11390-4)
Supplement: Supplementary file 2 — Additional file 2: Table 1. List of PD-L1/TGF-β dual antibody research and development. Table 2. summary of serum pharmacokinetic parameters after intravenous infusion of 1, 10 and 60 mg/kg TQB2858 in cynomolgus monkeys (n = 6). Table 3. Detection of anti-drug antibodies in cynomolgus monkeys after intravenous administration of TQB2858 Neutralizing activity detection rate. Figure 1. Mean plasma concentration-time curves of each group after intravenous injection of different doses of TQB2858 injection in cynomolgus monkeys. Table 4. Toxicokinetic parameters in 4-week repeated dose intravenous toxicity study in cynomolgus monkeys. Figure 2. Mean concentration-time curve of TQB2858 in serum of cynomolgus monkeys after intravenous injection of TQB2858. Table 5. List of specific laboratory test items. Table 6. Unlisted in NCI-CTCAE v5.0 Criteria for Judging the Severity of Adverse Events. Table 7. Form for Judging the Relationship between Adverse Event and Drug. [file 12885_2023_11390_MOESM2_ESM.pdf]

Registration classification: Class I therapeutic biological product

Single-arm, open-label, multicenter phase Ib clinical trial of TQB2858 injection in the treatment of advanced high-grade sarcoma

Protocol No.	TQB2858-Ib-02
Protocol Version No.	1.1
Protocol Version Date	25 Nov 2021
Study site	Peking University People's Hospital
Study director	Prof. Guo Wei
Sponsor	Chia Tai Tianqing Pharmaceutical Group
	Nanjing Shunxin Pharmaceutical Co., Ltd.

Confidentiality Statement

The information contained in this clinical trial protocol is confidential and is intended solely for the use of clinical investigators. Its copyright is owned by Chia Tai Tianqing Pharmaceutical Group Nanjing Shunxin Pharmaceutical Co., Ltd. It shall not be disclosed to any third party (group or individual) without prior written permission. If you hold this protocol without prior authorization, please contact CHIA TAI TIANQING Pharmaceutical Group Nanjing Shunxin Pharmaceutical Co., Ltd. in a timely manner, and return the protocol and its photocopies to the company.

Organization Information Page

This study is jointly undertaken by the clinical trial sponsor, conducting unit, data management unit, statistical analysis unit, and experimental testing unit.

Sponsor of clinical trial: Chia Tai Tianqing Pharmaceutical Group Nanjing Shunxin Pharmaceutical Co., Ltd.

Contact address: No.1099 Fuying Road, Jiangning District, Nanjing, Jiangsu (Jiangning Hi-tech Park)

Function: Initiate and apply for the study, provide the investigator's brochure, investigational drugs, study fund, organize, monitor, audit and develop the clinical trial protocol.

Main participants are as follows:

Position	Name	Telephone	E-mail
Project Leader	Guodong Wu	15694147236	wuguodong66@126.com
Medical Director	Lifan Tu	15062242635	tulifan2015@163.com

Clinical trial Responsible unit	Company name: Peking University People's Hospital<br>Contact address: No. 11, Xizhimen South Street, Xicheng District, Beijing<br>Functions: Recruitment of subjects, development of clinical trial protocol, organization and implementation of clinical trial, and acceptance of inspection by the regulatory authority at any time.<br>Principal Investigator: Wei Guo. Tel: 1 3701195504
Clinical trial data management/statistical analysis unit	Name of unit: Chia Tai Tianqing Pharmaceutical Group Co., Ltd.<br>Contact Address: No. 1099, Fuying Road, Jiangning District, Nanjing City, Jiangsu Province (Jiangning High-tech Park)<br>Function: Responsible for data management and statistical analysis<br>Contact Person: Yadong Miao. Tel: 18551674600

Protocol Signature Page

We have read and confirmed this protocol (Protocol Number: TQB2858-Ib-02 Version number: 1.1, date: 25 Nov 2021). I agree to perform the responsibilities related to the sponsor in accordance with Chinese laws, Declaration of Helsinki, Chinese GCP and this study protocol.

Sponsor of clinical trial: CHIA TAI TIANQING PHARMACEUTICAL GROUP Nanjing Shunxin Pharmaceutical Co., Ltd.

Xunqiang Wang		
Clinical Technical Lead<br>(Print)	Signature	Date of Signature<br>(DD/MMM/YYYY)

Protocol Signature Page

I will earnestly perform my duties and personally participate in or directly guide this clinical trial in accordance with current GCP regulations in China. I have read and confirmed this protocol (protocol number: TQB2858-Ib-02, version number: 1.1, version date: November 25, 2021). I agree to perform the responsibilities related to the principal investigator in accordance with the laws of China, Declaration of Helsinki, China GCP and this study protocol, including supervising all investigators to implement the study protocol and taking measures to implement the quality management of the clinical trial.

I agree to timely sign and receive relevant safety information of the clinical trial provided by the sponsor, and consider whether the subject's treatment is adjusted accordingly, communicate with the subject as soon as possible when necessary, and report suspected and unexpected serious adverse reactions provided by the sponsor to the Ethics Committee.

Study site: Peking University People's Hospital

**Wei Guo**		
Principal Investigator (Print)	Signature of Principal Investigator	Date of Signature (DD/MMM/YYYY)

Protocol Signature Page

I will earnestly perform my duties and personally participate in or directly guide this clinical trial in accordance with current GCP regulations in China. I have read and confirmed this protocol (protocol number: TQB2858-Ib-02, version number: 1.1, version date: November 25, 2021). I agree to perform the responsibilities related to the principal investigator in accordance with the laws of China, Declaration of Helsinki, China GCP and this study protocol, including supervising all investigators to implement the study protocol and taking measures to implement the quality management of the clinical trial.

I agree to timely sign and receive relevant safety information of the clinical trial provided by the sponsor, and consider whether the subject's treatment is adjusted accordingly, communicate with the subject as soon as possible when necessary, and report suspected and unexpected serious adverse reactions provided by the sponsor to the Ethics Committee.

Study sites:

Principal Investigator (Print)	Signature of Principal Investigator	Date of Signature (DD/MMM/YYYY)

VERSION HISTORY/REVISION HISTORY

Version No.	Version Date	Reason for Amendment Description and Summary of Changes
1.0	August 27, 2021	NA
1.1	25 Nov 2021	1. Delete the contents related to dynamics unrelated to this test;<br>2. Erratum, where the text and flowchart are inconsistent.

Abbreviation

Abbreviation	Full name in Chinese	Abbreviation	Full name in Chinese
ADA	Anti-drug antibody	ITT	Intention-to-treat analysis
ADCC	Antibody-dependent cytotoxicity	LVEF	Haze score
AUC	Area under the plasma concentration-time curve	MRSD	Maximum Recommended Starting Dose
AUCINF	Area under the concentration-time curve from the time of dosing to infinity	MRT	Mean residence time
AUCss	Area under the plasma concentration-time curve at steady state	MTD	Maximum tolerated dose
CD	Antigenic determinant	NK	Natural Killer Cells
CDC	Complement-dependent cytotoxicity	NYHA	New York Heart Association
CL	Apparent clearance	ORR	Objective response rate
CL/F	Oral clearance	OS	Overall survival
Cmax	Maximum plasma concentration	PBMC	Peripheral blood mononuclear cell
CR	Complete response	PCR	Polymerase chain reaction
CRF	Case Report Form	PD	Disease progression/pharmacodynamics
Cmax, ss	Steady-state peak concentration	PD-1	Programmed cell death-1
Cmin, ss	Trough concentration at steady state	PD-L1	Programmed cell death protein ligand-1
CTC AE	Common Terminology Criteria for Adverse Events	PFS	Progression-free survival
CTLA-4	Cytotoxic T-lymphocyte-associated protein 4	PI	Principal Investigator
DC	Dendritic cell	PK	Kinetics
DCR	Disease control rate	PPS	Per Protocol Set
DLT	Dose limiting toxicity	PR	Partial response
DOR	Duration of Response	Q3w	Every 3 weeks
EC50	Effective concentration, 50%	QTC	Corrected Q-T interval
ECOG	Eastern Cooperative Oncology Group	RECIST	Response Evaluation Criteria in Solid Tumors
ECRF	Electronic Case Report Form	RO	Receptor occupancy
EDC	Electronic Data Capture	SAE	Serious adverse event
ELISA	Enzyme-linked immunosorbent reaction	SD	Stable disease
FACS	Fluorescence flow cytometric sorting	SS	Safety Analysis Set
FAS	Full Analysis Set	T1/2	Half-life

Abbreviation	Full name in Chinese	Abbreviation	Full name in Chinese
HCV	Hepatitis C Virus	TCR	T-cell receptor
HIV	Human Immunodeficiency Virus	ULN	Upper limit of normal
IgG4	Immunoglobulin G-4	Vd	Apparent volume of distribution
IRECIST	Response Evaluation Criteria in Immune Solid Tumors		

Protocol Abstract

Study title	Single-arm, open-label, multicenter phase Ib clinical trial of TQB2858 injection in the treatment of advanced high-grade sarcoma
Protocol No.	TQB2858-Ib-02
Version No./Date	1.1/25 Nov 2021
Study site	Peking University People's Hospital
Study director	Prof. Guo Wei
Test objective	Primary objective<br>● To evaluate the preliminary efficacy of TQB2858 injection in patients with advanced high-grade sarcoma;<br>Secondary objectives<br>● To evaluate the safety of TQB2858 injection in patients with advanced high-grade sarcoma;<br>Exploratory objectives<br>● To explore biomarkers associated with treatment of TQB2858 injection.
Trial Endpoints	Primary endpoint<br>1) ORR based on RECIST 1.1 and iRECIST;<br>Secondary endpoints<br>1) Other efficacy indicators (based on RECIST 1.1 and iRECIST): PFS, DCR, DOR, OS;<br>2) Incidence of adverse events: occurrence of all adverse events (AEs), serious adverse events (SAEs) and treatment-related adverse events (TEAEs);<br>Exploratory Endpoints

	1) To detect the biomarkers related to TQB2858 injection treatment, such as the expression of CD68 in tumor tissues CD163, CD206, IRF8, CD3, CD8, PD-L1, CD20, CD56, B7-H3, CD47, CD4 etc., and evaluate the proportion of tertiary lymph nodes.
Test object	Subjects with advanced high-grade sarcoma
Sample Size	A total of 30 – 100 cases.<br>Cohort 1:10-30 patients with primary alveolar soft part sarcoma;<br>Cohort 2:10-30 patients with alveolar soft part sarcoma failing to respond to PD-1 therapy will be enrolled;<br>Cohort 3:10-40 patients will be enrolled for other subtypes (pleomorphic sarcoma, classical osteosarcoma, Ewing sarcoma, chondrosarcoma, dedifferentiated liposarcoma, etc.).
Test design	This study is a single-arm, open-label, multicenter phase Ib clinical trial
Inclusion criteria	Patients who meet all of the following inclusion criteria can be included in this trial:<br>1) Histologically confirmed unresectable, recurrent, or metastatic high-grade sarcoma;<br>2) Patients who previously received at least one line of treatment, with disease progression or intolerance during treatment, or disease progression after the end of treatment; (except for alveolar soft part sarcoma and clear cell sarcoma)<br>a) Cohort 1: Primary alveolar soft part sarcoma;<br>b) Cohort 2: alveolar soft part sarcoma failing PD-1 therapy;<br>c) Cohort 3: Other subtypes (pleomorphic sarcoma, classic osteosarcoma, Ewing sarcoma, chondrosarcoma, dedifferentiated liposarcoma, etc.)<br>3) Age: 18-70 years old;<br>4) ECOG score: 0 ~ 1;<br>5) Life expectancy of more than 3 months;

	6) Normal function of major organs, i.e. meeting the following criteria:<br>a) Hematology (not transfused and not receiving growth factor support before screening): hemoglobin (HGB) ≥ 80 g/L; neutrophil count (NEUT) ≥ 1.5 × 10^9/L; platelet count (PLT) ≥ 90 × 10^9/L;<br>b) Biochemistry: Alanine aminotransferase (ALT) and aspartate aminotransferase (AST) ≤ 2.5 × ULN (ALT and AST ≤ 5 × ULN for patients with primary hepatobiliary tumors or liver metastases); total bilirubin (TBIL) ≤ 1.5 × ULN (TBIL ≤ 3 × ULN for patients with Gilbert's syndrome); creatinine (CRE) ≤ 1.5 × ULN or creatinine clearance ≥ 60 ml/min;<br>c) Coagulation function: activated partial thromboplastin time (APTT), international normalized ratio (INR), prothrombin time (PT) ≤ 1.5 × ULN;<br>d) Left ventricular ejection fraction (LVEF) ≥ 50%;<br>7) Female patients of childbearing potential must have a negative serum or urine HCG test within 7 days prior to study enrollment and must be non-lactating; patients should agree to use contraception during the study and for 6 months after the end of the study period;<br>8) The patient voluntarily joined the study, signed the informed consent form, and had good compliance.
Exclusion Criteria	Patients with any of the following criteria will not be included in the trial:<br>1) Concomitant diseases and medical history:<br>a) History or presence of other malignancy within 2 years. Patients with the following two situations can be enrolled: Other malignant tumors treated by single surgery and can achieve R0 resection without recurrence and metastasis; Cured cervical carcinoma in situ, non-melanoma skin cancer, nasopharyngeal carcinoma and superficial bladder tumors [Ta (non-invasive tumor), Tis (carcinoma in situ) and

	T1 (tumor infiltrating basal lamina)];<br>b) Unresolved toxicity greater than CTCAE Grade 1 due to any prior therapy, excluding alopecia, peripheral sensory neuropathy;<br>c) Major surgical treatment or significant traumatic injury (excluding needle biopsy, endoscopic biopsy, etc.) within 28 days before the start of study treatment;<br>d) Wounds or fractures that have not been healed for a long time;<br>e) Hyperactive/venous thrombotic events within 6 months, such as cerebrovascular accident (including temporary ischemic attack, cerebral hemorrhage, cerebral infarction), deep venous thrombosis and pulmonary embolism;<br>f) Patients with a history of psychotropic drug abuse and unable to quit or with mental disorders;<br>g) Subjects with any severe and/or uncontrolled disease including:<br>1. Unsatisfactory blood pressure control (systolic blood pressure ≥ 150 mmHg or diastolic blood pressure ≥ 100 mmHg);<br>2. Patients with unstable angina pectoris/cardiac chest pain ≥ grade 2; myocardial infarction ≤ 12 months before enrollment; heart failure ≥ grade 1 (New York Heart Association (NYHA) classification); restrictive cardiomyopathy; atrioventricular block ≥ grade 2, arrhythmia not stably controlled by drugs [including QTc ≥ 450 ms (male), QTc ≥ 470 ms (female)] and arrhythmia that may have potential impact on the trial treatment;<br>3. Active infection (≥ CTCAE grade 2 infection);<br>4. Decompensated cirrhosis, active hepatitis *;

	* Active hepatitis (hepatitis B reference: HBsAg positive, and HBV DNA > 2500 copies/mL or > 500 IU/mL; hepatitis C reference: HCV antibody positive, and HCV virus test value exceeds the upper limit of normal); note: eligible subjects with hepatitis B surface antigen positive or core antibody positive, hepatitis C patients, continuous antiviral therapy is recommended to prevent viral activation.<br>5. Patients with renal failure requiring hemodialysis or peritoneal dialysis;<br>6. History of immunodeficiency, including HIV positive or suffering from other acquired, congenital immunodeficiency diseases, or history of organ transplantation;<br>7. Poorly controlled diabetes (fasting blood glucose (FBG) > 10 mmol/L);<br>8. Urinalysis showed urine protein ≥ + +, and confirmed 24-hour urine protein > 1.0 g;<br>9. Patients with epilepsy requiring treatment;<br>2) Tumor related symptoms and treatment:<br>a) Patients who previously received surgery, chemotherapy, radiotherapy or other anti-cancer therapy 4 weeks before the start of study treatment (the washout period will be calculated from the time of last treatment); patients who previously received local radiotherapy can be eligible if: the end of radiotherapy is more than 4 weeks from the start of study treatment (more than 2 weeks for brain radiotherapy); and the target lesion selected for this study is not in the radiotherapy area; or the target lesion is located in the radiotherapy area, but progression has been confirmed.

b) 2. Patients who have received Chinese patent medicines with anti-tumor indications (including compound cantharidin capsules, Kangai injection, Kanglaite capsules/injection, Aidi injection, Brucea javanica oil injection/capsules, Xiaoer Fangye/injection, cinobufotalin capsules, etc.) in the package insert of NMPA approved drugs before the start of study treatment;

c) Uncontrolled pleural effusion, pericardial effusion or ascites requiring repeated drainage (judged by the investigator);

d) Patients with brain metastasis whose symptom control is stable for less than 4 weeks after stopping dehydrating agents and steroid drugs;

3) Study Treatment Related:

a) History of live attenuated vaccination 28 days before the start of study treatment or planned live attenuated vaccination during the study;

b) Previous history of severe allergy to macromolecular drugs, or allergy to known components of TQB2858 injection;

c) Active autoimmune disease requiring systemic therapy (eg, use of disease-modifying drugs, corticosteroids, or immunosuppressive agents) within 2 years of the start of study treatment. Replacement therapy (e.g., thyroxine, insulin, or physiological corticosteroids for adrenal or pituitary insufficiency, etc.) is not considered systemic;

d) Diagnosis of immunodeficiency or ongoing systemic glucocorticoid therapy or any other form of immunosuppressive therapy. (Dose > 10 mg/day prednisone or other effective

	hormones), and the drug is still in use 2 weeks before the first dose;<br>4) Participated in other clinical trials of anti-tumor drugs 4 weeks before enrollment;<br>5) Patients with concomitant diseases that, in the judgment of the investigator, seriously endanger the safety of the subjects or affect the completion of the study, or are considered unsuitable for the subjects for other reasons;
Test drug	TQB2858 injection, specification: 200 mg (4 ml)/vial
Dosing Regimen	TQB2858 injection: once every 3 weeks (dosing window: ± 3 days), 3 weeks as a treatment cycle, until disease progression or investigator's judgment is not appropriate to continue the treatment. According to the determined RP2D dose, the given dose of TQB2858 was diluted with 0.9% sodium chloride injection [0.9% (w/v) sodium chloride solution, the same below] to a final protein concentration of 0.1 mg/ml ~ 10 mg/ml (such as 30-250 ml), and the entire contents of the intravenous infusion bag were intravenously infused into the body using an infusion set with an inline filter of 0.2 μm or 0.22 μm, with an infusion time of 20-60 ± 10 min. The infusion time starts from the start of infusion of TQB2858, ends at the end of infusion of TQB2858 and the end of flushing with 0.9% sodium chloride injection (it is recommended to flush with 20ml 0.9% sodium chloride injection).<br>In case of infusion reactions, appropriate intervention measures are recommended in "Recommendations for the Management of Immune-Related Adverse Events Caused by Immune Checkpoint Inhibitor Therapy" and "Chinese Society of Clinical Oncology (CSCO) Guidelines for the Management of Immune-Checkpoint Inhibitor-Related Toxicity 2019".
Biomarker study	To explore the expression of CD68 in tumor tissue CD163, CD206, IRF8, CD3, CD8, PD-L1, CD20, CD56, B7-H3, CD47, CD4 etc., and to

	determine the presence of tertiary lymph node structure in tumor tissues based on the positive results in the above tissue sections, it is planned to collect 6 white tablet of tumor tissues from subjects at different time points before and after administration in this study (one piece of 5 microns, for smear and baking glue, staining immunohistochemistry), to determine the characteristics of TQB2858 in the immunogenic microenvironment of subjects with advanced high-grade sarcoma.
Efficacy evaluation criteria	RECIST 1.1/iRECIST. The response assessment criteria for this study were based on RECIST 1.1, while iRECIST criteria were used to confirm response.
Safety evaluation criteria	The severity of adverse events was judged using NCI-CTCAE 5.0 criteria. Faithfully fill in the adverse event record form during the trial, including the occurrence time and severity of adverse events, correlation with the study treatment, duration, measures taken and outcome.
Data Analysis and Statistical Methods	**Efficacy Analysis**<br>➢ Primary endpoints:<br>Objective response rate (ORR): The percentage of subjects with complete response (CR) and partial response (PR) among evaluable efficacy subjects will be calculated, and the proportion of objective response cases (CR + PR) in the total number of cases will be calculated, with 95% confidence interval (CI). The 95% CI for ORR was calculated based on the exact binomial method using the F-distribution.<br>➢ Secondary endpoints:<br>Disease control rate (DCR): The percentage of subjects with complete response (CR), partial response (PR) and stable disease (SD) among evaluable efficacy subjects will be calculated, and the proportion of the number of cases with disease control (CR + PR + SD) in the total number of cases will be calculated, along with 95% confidence interval (CI). The 95% CI for DCR was calculated based on the exact binomial method using the F-distribution.

	Duration of response (DOR), overall survival (OS): Kaplan-Meier method was used to estimate DOR, OS and its 95% CI, and Kaplan-Meier curve was plotted.<br>**Safety analysis**<br>Including adverse events/adverse reactions, serious adverse events/serious adverse reactions, vital signs, laboratory tests, 12-Lead ECG, concomitant medication, etc. Based on SS, mainly descriptive statistics summary.<br>The number, percentage, and percentage of all adverse events, treatment-emergent adverse events (TEAEs), Grade 3 or higher TEAEs, Grade 3 or higher TEAEs related to study drug, treatment-emergent serious adverse events (SAEs), TEAEs related to study drug, SAEs related to study drug, TEAEs leading to treatment discontinuation, and TEAEs leading to death will be summarized by MedDRA dictionary by SOC and PT for each group.<br>Laboratory test results, vital signs, ECG and other data will be analyzed using conversion tables for baseline and post-baseline conditions.
Estimated Trial Schedule	Proposed start time: November 2021 (after approval by the Ethics Committee).<br>Estimated duration of trial: November 2021-November 2023.

TQB2858 Injection Phase Ib Trial Subject Visit Schedule

Item	Screening [1]	Treatment period										Follow-up period		
		Cycle 1				Cycle 2		Cycle 2n + 1		Cycle 2n + 2		Out Visit [2]	Safety follow-up [3]	Survival Follow-up [4]
		C1D1	C1D7	C1D14	C1D21	C2D1	C2D21	D1	D21	D1	D21	Early Withdrawal or D28	12 weeks after medication	Every 8 weeks
Check Window [21]	-	-	-	-	± 3 days	-	± 3 days	-	± 3 days	-	± 3 days	+ 7 days		
Informed Consent	X													
Height, weight [5]	X	X	X	X	X	X	X	X	X	X	X	X		
Demographics/Medical History [6]	X													
ECOG score	X						X				X	X		
Vital signs [7]	X	X	X	X	X	X	X	X	X	X	X	X		
Physical examination [8]	X		X	X	X		X		X		X	X		
Infectivity tests [9]	X													
Blood routine [10]	X		X	X	X		X		X		X	X		
Blood chemistry [11]	X		X	X	X		X		X		X	X		
Thyroid function [12]	X			X	X		X		X		X	X		

Item	Screening [1]	Treatment period										Follow-up period		
		Cycle 1				Cycle 2		Cycle 2n + 1		Cycle 2n + 2		Out Visit [2]	Safety follow-up [3]	Survival Follow-up [4]
		C1D1	C1D7	C1D14	C1D21	C2D1	C2D21	D1	D21	D1	D21	Early Withdrawal or D28	12 weeks after medication	Every 8 weeks
Check Window [21]	-	-	-	-	± 3 days	-	± 3 days	-	± 3 days	-	± 3 days	+ 7 days		
Coagulation [13]	X			X	X		X		X		X	X		
Urinalysis [14]	X		X	X	X		X		X		X	X		
Stool routine [15]	X		X	X	X		X		X		X	X		
Cardiac function [16]	X				X		X		X		X	X		
Echocardiogram	X													
Electrocardiogram [17]	X		X	X	X		X		X		X	X		
Imaging [18]	X						X				X	X		
Blood pregnancy test	X													
Study medication [19]		X				X		X		X				
Concomitant medication	X	X	X	X	X	X	X	X	X	X	X	X		
Adverse events	X	X	X	X	X	X	X	X	X	X	X	X		

Item	Screening [1]	Treatment period										Follow-up period		
		Cycle 1				Cycle 2		Cycle 2n + 1		Cycle 2n + 2		Out Visit [2]	Safety follow-up [3]	Survival Follow-up [4]
		C1D1	C1D7	C1D14	C1D21	C2D1	C2D21	D1	D21	D1	D21	Early Withdrawal or D28	12 weeks after medication	Every 8 weeks
Check Window [21]	-	-	-	-	± 3 days	-	± 3 days	-	± 3 days	-	± 3 days	+ 7 days		
Biomarker Sample collection [20]	X													

Notes:

1. Within 28 days after signing the informed consent form, it belongs to the screening period;
2. For details about the out-group visit, see Chapter 6 for details;
3. Safety visit: The safety follow-up period is the interval between the EOT visit and the scheduled follow-up visit, which should occur 30 days (± 7 days) after the EOT visit or 30 days (± 7 days) after study drug administration if the EOT visit was not performed.
4. Survival Visit: The first survival follow-up period (including telephone follow-up) will occur 8 weeks (± 7 days) after the last safety follow-up and occur every 8 weeks (± 7 days) until the subject has died, is lost to follow-up, the sponsor terminates the study, or other study closure criteria are met, whichever occurs first.
5. Height and weight: Height will be measured only during the screening period;
6. Demographics/medical history: Demographic data include date of birth, age, gender, ethnicity and occupation; medical history includes previous treatment regimen, concomitant diseases and treatment, allergy history and smoking history;

7. Vital signs: including body temperature, pulse, respiration and blood pressure;
8. Physical examination: see Chapter 6 for details;
9. Infectivity test: hepatitis B virus, HCV and HIV; if HBsAg or HBeAg is positive, HBV DNA test should be performed; if HCV antibody is positive, HCV RNA test should be performed;
10. Blood routine: See Table 5 for details;
11. Blood biochemistry: See Table 5 for details;
12. Thyroid function: See Table 5 for details;
13. Coagulation function: See Table 5 for details;
14. Urinalysis: see Table 5 for details;
15. Stool routine: see Table 5 for details;
16. Cardiac function: See Table 5 for details;
17. ECG: If chest pain, palpitation and other symptoms occur, echocardiography, myocardial enzyme spectrum and troponin should be additionally examined;
18. Imaging examination: see 6.3 for details;
19. Study drug: 3-week treatment cycle, administered on Day 1 of each cycle via intravenous drip, q3w; see details 5.4 Section content ;
20. Biomarkers: see Chapter 7 for details ;

Check window period: the time window allowed for the visit on the specified visit day. If the specific item has the time window requirement, implement it according to the specific item requirement

1. Test background

1.1 Foreword

Immunotherapy is a research hotspot of tumor immunology at present. Through the inhibition of immune checkpoints, the reactivation of immune cells is realized to avoid the immune escape of tumor cells, so as to kill tumor cells. In recent years, great achievements have been made in the study of some immune checkpoints, such as CTLA-4 and PD-1/PD-L1. Cancer immunotherapy can be described as blooming everywhere, with a wide range of indications, and various solid tumors and hematological tumors have corresponding immunotherapy studies. However, there are still shortcomings in cancer immunotherapy, such as the inability of certain patients from Immune checkpoint inhibitor therapy Benefit even in the indicated population of immune checkpoint inhibitors. In addition, some patients may benefit from starting immune checkpoint inhibitor therapy, but their tumors progress quickly. These shortcomings mean that there is still a way to go for cancer immunotherapy, and more tumor immune escape mechanisms await exploration.

Programmed death factor 1 and its ligand (PD-1/PD-L1) are a pair of immune costimulatory factors. Normally, PD-1 plays an immunoregulatory role through its ligand PD-L1. In recent years, PD-1 and its ligand PD-L1 have received attention because of their involvement in tumor immune escape mechanisms. Activation of PD-1/PD-L1 signaling pathway can lead to the formation of immunosuppressive tumor microenvironment, allowing tumor cells to evade immune surveillance and killing, while blocking PD-1/PD-L1 signaling pathway can reverse tumor immune microenvironment and enhance endogenous anti-tumor immune effect [1]. PD-L1 is highly expressed in various solid malignant tumors, including cancer, melanoma, renal cell carcinoma, prostate cancer, breast cancer, and glioma, and its expression levels vary according to tumor type [2]It can not only promote the growth of tumor cells, but also induce the apoptosis of T lymphocytes.

Transforming growth factor-β (TGF-β) belongs to a superfamily of cytokines that

promote cell growth and transformation. At present, a total of five isoforms of TGF-β are found, of which three are found in mammals: TGF-β1, TGF-β2, and TGF-β3. TGF-β is widely present in various normal and transformed cells, such as platelets, monocytes, macrophages, lymphocytes and fibroblasts. Almost all cells contain receptors for TGF-β. TGF-β has 3 main cellular receptors: type I, type II, and type III receptors. TGF-β is an important cytokine in organisms and has a variety of biological effects, including embryonic development, wound healing, chemotaxis and cell cycle regulation. Epithelial-mesenchymal transition (EMT) is the most important way for tumor cells to acquire invasion and metastasis, and TGF-β is a key factor known to induce EMT in tumor cells and plays a very important role in tumor occurrence and metastasis [3].

PD-L1/TGF-β double antibody binds both PD-L1 and TGF-β1 and improves the immune microenvironment by inhibiting the TGF-β pathway, thereby enhancing the efficacy of immunotherapy. At present, Merck's M7824 has achieved good results in phase I clinical studies and is currently in phase II clinical trials.

The following table shows PD-L1/TGF- at home and abroad Beta Development of dual antibodies.

Table 1List of PD-L1/TGF-β dual antibody research and development

Drug	Company	R & D Status
M7824	Merck KGaA/GSK	Phase 2
SHR1701	Hengrui	Phase 2

Soft tissue sarcomas (STS) and osteosarcomas are malignant tumors of mesenchymal origin with more than 50 subtypes and rare onset. Sarcomas are one of the five leading causes of cancer death in people under 20 years of age. Current treatments include surgery, radiotherapy, chemotherapy, and comprehensive treatment, while patients with advanced disease mainly rely on systemic drug therapy. Sarcomas have special immunological characteristics, so immunotherapy also has relevant basic and clinical research results in its comprehensive treatment. Studies have found that immunotherapy methods such as programmed cell death-1 (PD-1), programmed cell

death ligand 1 (PD-L1) and cytotoxic T lymphocyte-associated antigen 4 (CTLA-4) inhibitors play an important role in the treatment of various tumors such as malignant melanoma.

Pembrolizumab, in clinical studies with SARC028, included STS patients over 18 years of age or osteosarcoma patients over 12 years of age with histological evidence of metastatic or surgically unresectable locally advanced sarcoma that could have received up to 3 prior lines of therapy. Pembrolizumab 200 mg intravenously every 3 weeks for the primary endpoint of objective response rate (ORR). Of the 80 evaluable patients, only 7 (18%) of 40 STS patients had an objective response to pembrolizumab treatment, including 4 (40%) of 10 undifferentiated pleomorphic sarcomas, 2 (20%) of 10 liposarcomas, 1 (10%) of 10 synovial sarcomas, and none of 10 leiomyosarcomas. Among 40 cases of osteosarcoma, only 2 cases (5%) had objective response, including 22 cases of osteosarcoma in 1 case (5%), 5 cases of chondrosarcoma in 1 case (20%), and 13 cases of Yuvenn's sarcoma did not respond to treatment. Soft tissue sarcoma cohort, ORR 18%, mDOR 33 weeks, mPFS 18 weeks, PFS rate 55% at 12 weeks, mOS 49 weeks; undifferentiated pleomorphic sarcoma mPFS 30 weeks, PFS rate 70% at 12 weeks; liposarcoma mPFS 25 weeks, PFS rate 60% at 12 weeks [4].

Sarcoma subtypes are numerous, with high recurrence rate and poor prognosis. With the gradual deepening of the understanding of sarcoma tissue and biological behavior, immunotherapy will become a new therapeutic hope for sarcoma in the future.

1.2 Name, pharmacological type and mechanism of action of study drug

1.2.1 Test drug

Generic name of drug: TQB2858 injection

English name: TQB2858 Solution for Injection

Pinyin: TQB2858 Zhusheye

Formerly known as: TQB2814 Injection

Molecular formula: $C_{7862}H_{12150}N_{2090}O_{2484}S_{78}$ (no post-translational modifications)

Molecular weight: 178162 Da (heavy chain N-terminal cyclized, non-glycosylated)

The active ingredient of this product, TQB2858, is a humanized IgG1 antibody that targets both PD-L1 and TGFβ. It is composed of two light and two heavy chains of PD-L1 and the extracellular domain of TGFβRII.

1.2.2 Pharmacological type

TQB2858 is an anti-PD-L1/TGF-β bifunctional fusion protein.

1.2.3 Mechanism of Action

TQB2858 is a bifunctional fusion protein that is a PD-L1/TGF-β double antibody and consists of two parts. At one end is the antibody structure (Y) that can recognize and bind PD-L1, which is the PD-L1 antibody of our company's TQB2450 and is in clinical phase III; at the other end is the TGF-β receptor type II fusion protein (Trap) that can bind TGF-β and can capture TGF-β, thereby reducing TGF-β in and around tumor tissue. Given this unique mechanism of action, TQB2858 is an upgraded version of PD-L1.

Members of the CD28 superfamily of programmed death-1 (PD-1) are expressed in activated T cells, B cells, and myeloid cells, and they have two ligands, namely programmed cell death ligand 1 (PD-L1) and PD-L2. PD-L1 is expressed on a variety of tumor tissues and binds to the receptor PD-1 on T cells to negatively regulate immune responses. Several factors can induce the expression of PD-L1 on tumor cells, and the simultaneous expression of PD-L1 facilitates the occurrence and growth of tumors. PD-1/PD-L1 pathway inhibitors can block the binding of PD-1 to PD-L1, block negative regulatory signals, so that T cells can recover their activity, thereby enhancing the immune response. Therefore, immune regulation targeting PD-1/PD-L1 is of great significance for tumor inhibition.

Despite breakthroughs in cancer immune checkpoint blockade therapy, only 20 – 30% of patients are able to respond continuously, 50% are primary resistant, and 20 – 30% are secondary resistant. Therefore, a variety of treatment approaches are used for drug-resistant patients, such as: using chemotherapy, oncolytic viral drugs to further activate the immune system, or targeting the tumor stroma (using PD-L1/TGF-β double

antibody) to regulate the tumor microenvironment, as well as acting on a variety of immune cells at the same time, finding out the "intervention point" from many aspects to improve the cure rate of patients.

Transforming growth factor-β (TGF-β) belongs to the TGF-β superfamily that regulates cell growth and differentiation. TGF-β signals through heterotetrameric receptor complexes to promote tumor invasion and metastasis by mediating the SMAD pathway, while inhibiting the activation and differentiation of T cells and increasing PD-L1 expression, which in turn promotes tumor growth.

Compared with PD-L1 treatment, the use of PD-L1/TGF-β dual antibody can increase intratumoral CD8 + T cells and reduce immunosuppressive myeloid cells, making tumors more susceptible to PD- (L) 1 checkpoint blockade. Moreover, this product is targeted to capture free TGF1 and avoid the dose-limiting toxicity of pan-TGF-β inhibitors, such as cardiotoxicity and valvular heart disease. Therefore, inhibition of PD-1/PD-L1 pathway on the basis of TGF-β targeting and neutralizing the tumor microenvironment can restore the activity of T cells, enhance the immune response, and more effectively improve the effect of inhibiting tumor development and progression.

1.3 Non-clinical study results

1.3.1 Pharmacological study

1.3.1.1 Primary pharmacodynamics

Pharmacologically, in vitro pharmacodynamic and in vivo pharmacodynamic studies were performed. The in vitro pharmacodynamic results showed that this product selectively binds to human TGFβ and human PD-L1, significantly inhibits TGFβ1-stimulated luciferase reporter gene expression, and significantly promotes the secretion of IFN-γ by CD4 + T cells in SEB-stimulated or MLR; there was no significant ADCC; after incubation with PBMCs in vitro, the cytokine production of this product was comparable to that of hIgG1 in the isotype control group. Overall, the in vitro activity of this product on TGFβ was comparable to that of IgG1-TGFβ, and the in vitro activity

on PD-L1 was comparable to that of TQB2450. The in vivo pharmacodynamic results showed that this product significantly inhibited the growth of mouse colon cancer MC38/hPD-L1 subcutaneous xenograft in a dose-dependent manner.

1.3.1.2 Safety Pharmacology Studies

The safety pharmacology test was accompanied by a 4-week repeated dose toxicity test in cynomolgus monkeys to investigate the effects of intravenous injection of this product on the body temperature, respiratory rate and cardiovascular system of cynomolgus monkeys. The results showed that intravenous injection of this product in cynomolgus monkeys had no damaging effect on their body temperature, respiratory rate and cardiovascular system.

1.3.2 Toxicology Studies

1) Drug toxicity study

During the study, no death or moribundity of animals was observed, and no abnormalities were observed in various indicators. The maximum tolerated dose (MTD) of intravenous injection in cynomolgus monkeys was 1000 mg/kg.

2) Repeat-Dose Toxicity Studies

4-week toxicity test of intravenous injection in cynomolgus monkeys

A total of 40 cynomolgus monkeys were used in this study and divided into 4 groups, 10 monkeys in each group (F: M = 1:1): control group (TQB2858 injection (blank)) and TQB2858 20, 60 and 200 mg/kg groups. Once weekly for 4 consecutive weeks (5 doses in total). For dosing groups, toxicokinetic blood samples were collected before the first (Day 1) dosing and at 0, 3, 8, 24, 48, 72, 120 and 168 hours after the end of dosing, before the 3rd and 4th (Days 15 and 22) dosing and at 0 hours after the end of dosing, and before the 5th (Day 29) dosing and at 0, 3, 8, 24, 48, 72, 120, 168, 216, 264, 336, 504, 672 and 1008 hours after the end of dosing. Immunogenicity was measured at 0 h before administration, 336 h and 672 h after administration, and on days 7, 14, and 28 of the recovery period in the treatment group. The plasma concentration and time change of this product in cynomolgus monkey serum were

determined by ELISA, and the immunogenicity of anti-this product antibody in serum was determined by ELISA. All methods were methodologically validated.

Decreased peripheral blood RBC, HGB and HCT and increased RET and RET% were observed in the 60 and 200 mg/kg groups, and mild increase in the proportion of red blood cells and late erythroblasts in the bone marrow smear was also observed in the 200 mg/kg group. The above changes were related to the pharmacological action of this product. By the end of recovery period, the above indicators of monkeys in each dose group showed recovery to varying degrees. At the end of treatment, minimal mononuclear cell infiltration in thyroid gland was observed in ≥ 20 mg/kg group, minimal ~ mild mononuclear cell infiltration in kidney was observed in ≥ 60 mg/kg group, and minimal mononuclear cell infiltration in meninges was observed in 20 mg/kg and 200 mg/kg groups, which were adverse reactions related to pharmacological effects. Similar reports were also found in the study data of marketed PD-L1 monoclonal antibody. At the end of recovery period, except that meningeal lesions in 200 mg/kg group showed no significant recovery, other organ lesions showed recovery to some extent. After intravenous infusion in cynomolgus monkeys, anti-drug antibodies were detected after drug administration in 9 (9/10), 9 (9/10) and 3 (3/10) animals in each dose group, respectively.

In addition, no significant abnormal changes were observed in general observation, body weight, food consumption, body temperature, ECG, respiratory rate, blood pressure, blood biochemistry, urine, ophthalmological examination, bone marrow smear, immunoglobulin, complement, circulating immune complexes, lymphocytes and cytokines in all groups.

In conclusion, under the conditions of this study, 20, 60 and 200 mg/kg TQB2858 injection was intravenously injected to cynomolgus monkeys once weekly for 4 consecutive weeks (5 times), followed by a 6-week recovery period. The highest non-severely toxic dose (HNSTD) was 200 mg/kg, and the exposure was 210 ± 40.8 and 209 ± 86.7 hmg/mL in male and female monkeys, respectively.

3) Hemolysis and vascular irritation test

This product has no hemolysis and vascular irritation reaction.

1.3.3 Studies on dynamics

Kinetic test of intravenous injection in cynomolgus monkeys

A total of 18 cynomolgus monkeys were used in this study and divided into 3 groups, 6 monkeys in each group, half males and half females, which were intravenously infused with 1, 10, and 60 mg/kg of this product, respectively. In treatment group, blood samples were collected at 0 h before administration and 1 min, 3 h, 8 h, 24 h, 48 h, 72 h, 120 h, 168 h, 216 h, 264 h, 336 h, 504 h and 672 h after the start of administration to detect plasma concentrations. Immunogenicity was measured at 0 h before administration and 168 h, 336 h, and 672 h after administration in the treatment group. The plasma concentration and time change of this product in cynomolgus monkey serum were determined by ELISA, and the immunogenicity of anti-this product antibody in serum was determined by ELISA. All methods were methodologically validated.

Table 2. summary of serum pharmacokinetic parameters after intravenous infusion of 1, 10 and 60 mg/kg TQB2858 in cynomolgus monkeys (n = 6)

Parameter	Units	TQB2858								
		1 mg/kg			10 mg/kg			60 mg/kg		
		Female	Male	Mean value	Female	Male	Mean value	Female	Male	Mean value
AUCINF obs	h* μg/mL	444 ± 164	460 ± 162	452 ± 146	8150 ± 311	8270 ± 558	8210 ± 410	44500 ± 10500	55200 ± 10200	49800 ± 10900
AUClast	h* μg/mL	434 ± 170	447 ± 165	441 ± 150	8040 ± 341	7980 ± 551	8010 ± 411	44200 ± 11000	55000 ± 10400	49600 ± 11200
Clobs	mL/hr/kg	2.47 ± 0.924	2.42 ± 1.04	2.45 ± 0.879	1.23 ± 0.0462	1.21 ± 0.0850	1.22 ± 0.0618	1.41 ± 0.383	1.11 ± 0.195	1.26 ± 0.316
Cmax	Mg/mL	20.6 ± 1.51	19.2 ± 0.819	19.9 ± 1.33	214 ± 16.0	220 ± 13.5	217 ± 13.7	1360 ± 80.8	1260 ± 72.3	1310 ± 90.1
MRTINF obs	h	35.2 ± 12.0	43.1 ± 10.2	39.2 ± 10.8	66.7 ± 3.50	81.2 ± 8.44	74.0 ± 9.85	84.0 ± 11.6	109 ± 9.98	96.6 ± 16.9
MRTlast	h	32.1 ± 13.5	38.1 ± 11.8	35.1 ± 11.8	63.5 ± 2.86	72.5 ± 6.54	68.0 ± 6.69	80.1 ± 15.6	107 ± 7.39	93.8 ± 18.5
T1/2	h	26.5 ± 7.55	33.2 ± 5.97	29.8 ± 7.11	42.0 ± 7.06	36.7 ± 31.1	39.3 ± 20.4	56.1 ± 34.4	66.6 ± 38.9	61.3 ± 33.4
Tmax	h	0.0167 ± 0.00	0.0167 ± 0.00	0.0167 ± 0.00	0.0167 ± 0.00	0.0167 ± 0.00	0.0167 ± 0.00	0.0167 ± 0.00	0.0167 ± 0.00	0.0167 ± 0.00
Vssobs	mL/kg	80.0 ± 2.87	97.3 ± 15.4	88.6 ± 13.7	81.8 ± 2.95	98.2 ± 8.43	90.0 ± 10.6	116 ± 22.5	122 ± 29.0	119 ± 23.5

Table 3 Detection of anti-drug antibodies in cynomolgus monkeys after intravenous administration of TQB2858 Neutralizing activity detection rate

Group	Dose Frequency	Dose (mg/kg)	Anti-drug antibody detection rate
TQB2858	Once Intravenously	1	6/6
	Once Intravenously	10	6/6
	Once Intravenously	60	4/6

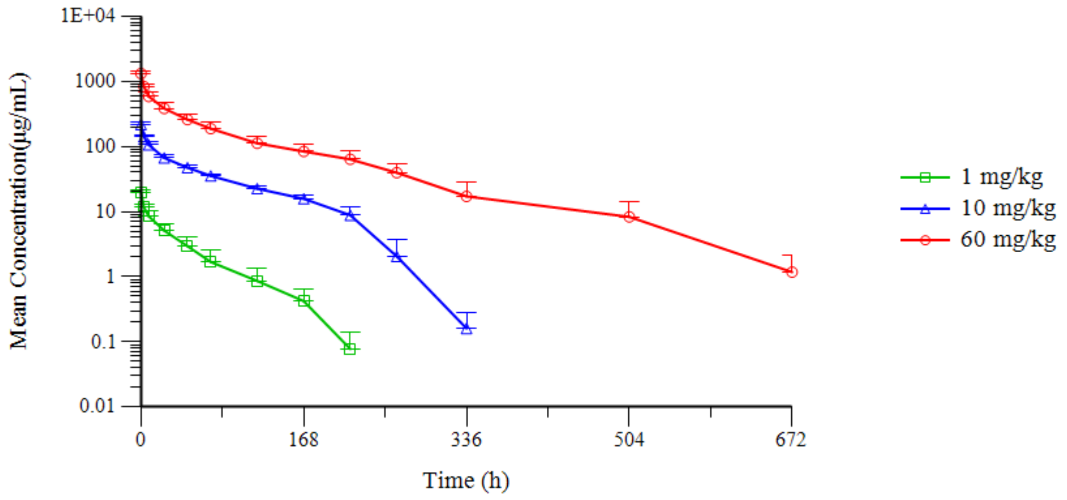

Figure 1 Mean plasma concentration-time curves of each group after intravenous injection of different doses of TQB2858 injection in cynomolgus monkeys

After intravenous infusion of 1, 10 and 60 mg/kg TQB2858, the serum drug concentration level increased with the increase of dose, and there was no significant gender difference in the main kinetic parameters. The terminal elimination half-life T1/2 was 29.8 ± 7.11 h, 39.3 ± 20.4 h and 61.3 ± 33.4 h, Cmax was 19.9 ± 1.33 h, 217 ± 13.7 h and 1310 ± 90.1 μg/mL, AUCINFobs was 452 ± 146 h * μg/mL, 8210 ± 410 h * μg/mL and 49800 ± 10900 h * μg/mL, respectively. The dose ratio was 1:10:60, the proportional relationship of Cmax was 1:10.90:65.83 and the proportional relationship

of AUCINFobs was 1:18.16:110.18 in each group. The increase of Cmax in Cynomolgus monkeys was directly proportional to the increase of dose. In the range of 1 ~ 10 mg/kg, the exposure (AUC) of this product in serum increased slightly more than dose-proportionally; in the range of 10 ~ 60 mg/kg, the exposure increased basically in accordance with the dose-proportionally.

The results of ADA assay showed that after intravenous infusion in cynomolgus monkeys, ADA was detected in 6 (6/6 ratio), 6 (6/6 ratio) and 4 (4/6 ratio) animals in each dose group.

Repeated dose kinetic test in cynomolgus monkeys via intravenous injection

Because the doses were similar, the repeated dose kinetic test was accompanied by a 4-week continuous dose long-term toxicity test in cynomolgus monkeys.

A total of 40 cynomolgus monkeys were used in this study and divided into 4 groups, 10 monkeys in each group (F: M = 1:1): control group (TQB2858 injection (blank)) and TQB2858 20, 60 and 200 mg/kg groups. Once weekly for 4 consecutive weeks (5 doses in total). For dosing groups, toxicokinetic blood samples were collected before the first (Day 1) dosing and at 0, 3, 8, 24, 48, 72, 120 and 168 hours after the end of dosing, before the 3rd and 4th (Days 15 and 22) dosing and at 0 hours after the end of dosing, and before the 5th (Day 29) dosing and at 0, 3, 8, 24, 48, 72, 120, 168, 216, 264, 336, 504, 672 and 1008 hours after the end of dosing. Immunogenicity was measured at 0 h before administration, 336 h and 672 h after administration, and on days 7, 14, and 28 of the recovery periods in the treatment group. The plasma concentration and time change of this product in cynomolgus monkey serum were determined by ELISA, and the immunogenicity of anti-this product antibody in serum was determined by ELISA. All methods were methodologically validated.

Table 4. Toxicokinetic parameters in 4-week repeated dose intravenous toxicity study in cynomolgus monkeys

Dose (mg/kg)	Time	Gender	Cmax (mg/mL)	**AUC_{0-168h} (hmg/mL)**
20	First	Female	0.467 ± 0.0911	15.9 ± 3.97
		Male	0.484 ± 0.116	17.4 ± 5.40
	Last	Female	0.282 ± 0.222	4.74 ± 6.53
		Male	0.366 ± 0.284	15.3 ± 17.4
60	First	Female	1.36 ± 0.126	43.9 ± 8.56

		Male	1.43 ± 0.212	52.8 ± 13.0
	Last	Female	1.48 ± 0.398	45.8 ± 17.0
		Male	1.25 ± 0.459	35.9 ± 19.7
200	First	Female	5.96 ± 0.640	176 ± 31.2
		Male	4.42 ± 0.772	168 ± 35.4
	Last	Female	5.50 ± 1.32	209 ± 86.7
		Male	5.62 ± 1.11	210 ± 40.8

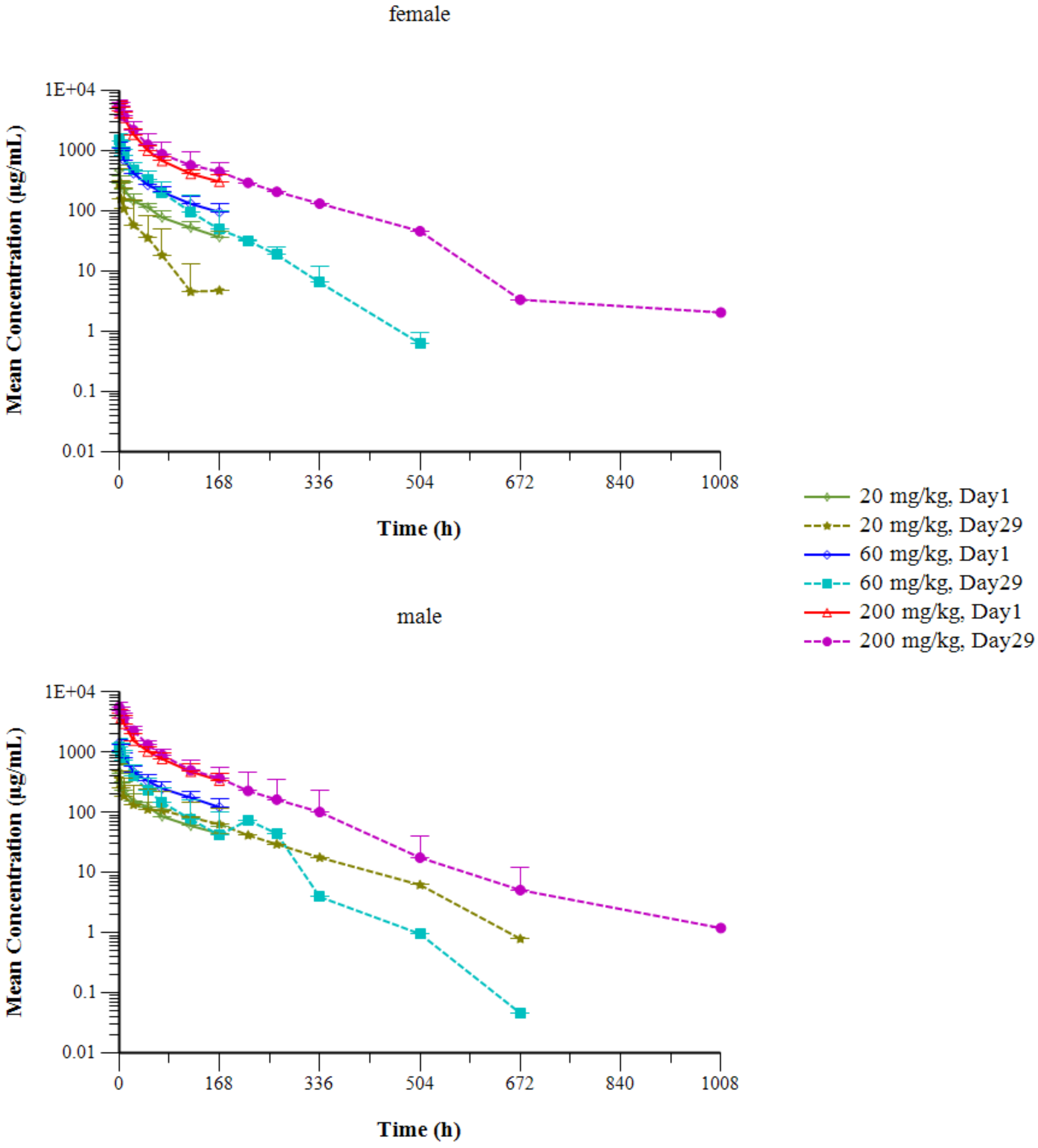

Figure 2 Mean concentration-time curve of TQB2858 in serum of cynomolgus monkeys after intravenous injection of TQB2858

After the first administration, the mean serum AUC_{0-168h} of female monkeys in each group was 15.9, 43.9 and 176 h mg/mL, respectively, with a ratio of 1:2.8:11.1;

the mean serum AUC_{0-168h} of male monkeys was 17.4, 52.8 and 168 h mg/mL, respectively, with a ratio of 1:3.0:9.7. The mean AUC_{0-168h} ratios of this product in the serum of female and male monkeys in each group were 0.9, 0.8, and 1.0. The mean AUC_{0-168h} of TQB2858 in the serum of female monkeys in each group was 4.74, 45.8 and 209 hmg/mL, respectively, with a ratio of 1:9.7:44.1; the mean AUC_{0-168h} of TQB2858 in the serum of male monkeys was 15.3, 35.9 and 210 hmg/mL, respectively, with a ratio of 1:2.3:13.7. The mean AUC_{0-168h} ratios of this product in the serum of female and male monkeys in each group were 0.3, 1.3, and 1.0. Compared with the first dose, the mean AUC_{0-168h} ratios of this product in the serum of female monkeys in each group were 2.6, 2.1, and 2.0, respectively, and those of male monkeys were 2.7, 2.2, and 2.1, respectively. Compared with the first dose, the mean individual AUC_{0-168h} ratios of TQB2858 in serum were 0.3, 1.0 and 1.2 in female monkeys and 1.2, 0.7 and 1.3 in male monkeys in each group, respectively.

In the dose range of 20 ~ 200 mg/kg, this product was immunogenic to cynomolgus monkeys; except that the mean AUC_{0-168h} in female monkeys in the 20 mg/kg group was lower than that in male monkeys, there was no significant gender difference at the other doses after the first and last doses; except that the mean AUC_{0-168h} in female monkeys in the 20 ~ 60 mg/kg dose range increased slightly more than dose proportionally, the mean AUC_{0-168h} in monkeys in the first and last doses increased basically in accordance with dose proportionally. No significant accumulation was seen. After intravenous infusion in cynomolgus monkeys, anti-drug antibodies were detected after drug administration in 9 (9/10), 9 (9/10) and 3 (3/10) animals in each dose group, respectively. The production of anti-drug antibodies affected the evaluation of sex difference and accumulation index.

1.4 Advance in Clinical Research

The phase I study TQB2858-I-01 (CDE clinical trial registration number CTR20210612) of TQB2858 injection was conducted in Jilin Cancer Hospital. The study was formally initiated on April 12, 2021, and the first subject was completed on April 20, 2021. By July 1, 2021, a total of 6 subjects have been enrolled, including 1 subject in the 3 mg/human dose group, 1 subject in the 60 mg/human dose group, and

4 subjects in the 600 mg/human dose group (1 subject dropped out due to personal reasons, and 1 subject was added). At present, there are two CTCAE grade 1 rashes in the 600 mg/person dose group, which can subside spontaneously without the need for drug intervention. The rest had no adverse events. It suggests good safety. The 1200 mg/person dose group has now been initiated.

2. Trial objectives and endpoints

2.1 Primary objective

- To evaluate the preliminary efficacy of TQB2858 injection in subjects with advanced high-grade sarcoma.

2.2 Secondary objectives

- Evaluation the safety of TQB2858 injection in subjects with advanced high-grade sarcoma;

2.3 Exploratory objectives

- To explore biomarkers associated with treatment of TQB2858 injection.

2.4 Primary Endpoints

- ORR based on RECIST 1.1 and iRECIST;

2.5 Secondary Endpoints

- Other efficacy indicators (based on RECIST 1.1 and iRECIST): 3-month ORR, PFS, DCR, DOR, OS;
- Incidence of adverse events: occurrence of all adverse events (AEs), serious adverse events (SAEs) and treatment-related adverse events (TEAEs);

2.6 Exploratory Endpoints

To detect the biomarkers related to TQB2858 injection treatment, such as the expression of CD68, C D163 , C D206 , I RF8 , C D3 , C D8 , P D-L1 , C D20 , C D56 , B 7-H3 , C D47 , C D4 etc. in tumor tissues, and the proportion of tertiary lymph nodes was evaluated.

3. Study Design

This Phase Ib clinical trial was conducted based on the RP2D determined in the

Phase I dose escalation and cohort expansion clinical study of TQB2858 injection. It is planned to enroll 30-100 patients with advanced high-grade sarcoma (Cohort 1:10-30 patients with primary alveolar soft part sarcoma; Cohort 2:10-30 patients with alveolar soft part sarcoma failing PD-1 therapy; Cohort 3:10-40 patients with other subtypes (pleomorphic sarcoma, classical osteosarcoma, Yuventus sarcoma, chondrosarcoma, dedifferentiated liposarcoma, etc.)). To assess the preliminary efficacy and safety of TQB2858 injection in subjects with advanced high-grade sarcoma.

4. Subject Selection and Withdrawal

4.1 Inclusion criteria of subjects

Patients who meet all of the following inclusion criteria can be included in this trial:

1) Histologically confirmed unresectable, recurrent, or metastatic high-grade sarcoma;
2) Patients who previously received at least one line of treatment, with disease progression or intolerance during treatment, or disease progression after the end of treatment; (except for alveolar soft part sarcoma and clear cell sarcoma)
   a) Cohort 1: Primary alveolar soft part sarcoma;
   b) Cohort 2: alveolar soft part sarcoma failing PD-1 therapy;
   c) Cohort 3: Other subtypes (pleomorphic sarcoma, classic osteosarcoma, Ewing sarcoma, chondrosarcoma, dedifferentiated liposarcoma, etc.)
3) Age: 18-70 years old;
4) ECOG score: 0 ~ 1;
5) Life expectancy of more than 3 months;
6) Normal function of major organs meeting the following criteria:
   a) Hematology (not transfused and not receiving growth factor support before screening): hemoglobin (HGB) ≥ 80 g/L; neutrophil count (NEUT) ≥ 1.5 × 109/L; platelet count (PLT) ≥ 90 × 109/L;
   b) Biochemistry: alanine aminotransferase (ALT) and aspartate aminotransferase (AST) ≤ 2.5 × ULN (ALT and AST ≤ 5 × ULN for patients with primary hepatobiliary tumors or liver metastases); total bilirubin (TBIL) ≤ 1.5 × ULN

(TBIL ≤ 3 × ULN for patients with Gilbert's syndrome); creatinine (CRE) ≤ 1.5 × ULN or creatinine clearance ≥ 60 ml/min;

c) Coagulation function: activated partial thromboplastin time (APTT), international normalized ratio (INR), prothrombin time (PT) ≤ 1.5 × ULN;

d) Left ventricular ejection fraction (LVEF) ≥ 50%;

7) Female patients of childbearing potential must have a negative serum or urine HCG test within 7 days prior to enrollment and must be non-lactating; patients should agree to take contraceptive measures during the study and within 6 months after the end of the study period;

8) The patient voluntarily joined the study, signed the informed consent form, and had good compliance.

4.2 Exclusion criteria for subjects

Patients with any of the following criteria will not be included in the trial:

1) Concomitant diseases and medical history:

a) History or presence of other malignancy within 2 years. Patients with the following two situations can be enrolled: Other malignant tumors treated by single surgery can achieve R0 resection without recurrence and metastasis; Cured cervical carcinoma in situ, non-melanoma skin cancer, nasopharyngeal carcinoma and superficial bladder tumors [Ta (non-invasive tumor), Tis (carcinoma in situ) and T1 (tumor infiltrating basal lamina)];

b) Unresolved toxicity greater than CTCAE Grade 1 due to any prior therapy, excluding alopecia, peripheral sensory neuropathy;

c) Major surgical treatment or significant traumatic injury (excluding needle biopsy, endoscopic biopsy, etc.) within 28 days before the start of study treatment;

d) Wounds or fractures that have not been healed for a long time;

e) Hyperactive/venous thrombotic events within 6 months, such as cerebrovascular accident (including temporary ischemic attack, cerebral hemorrhage, cerebral infarction), deep venous thrombosis and pulmonary embolism;

f) Patients with a history of psychotropic drug abuse and unable to quit or with mental disorders;

g) Subjects with any severe and/or uncontrolled disease including:

1. Unsatisfactory blood pressure control (systolic blood pressure ≥ 150 mmHg or diastolic blood pressure ≥ 100 mmHg);
2. Patients with unstable angina pectoris/cardiac chest pain ≥ grade 2; myocardial infarction ≤ 12 months before enrollment; heart failure ≥ grade 1 (New York Heart Association (NYHA) classification); restrictive cardiomyopathy; atrioventricular block ≥ grade 2, arrhythmia not stably controlled by drugs [including QTc ≥ 450 ms (male), QTc ≥ 470 ms (female)] and arrhythmia that may have potential impact on the trial treatment;
3. Active infection (≥ CTCAE grade 2 infection);
4. Decompensated cirrhosis, active hepatitis *;
   * Active hepatitis (hepatitis B reference: HBsAg positive, and HBV DNA > 2500 copies/mL or > 500 IU/mL; hepatitis C reference: HCV antibody positive, and HCV virus test value exceeds the upper limit of normal); note: eligible subjects with hepatitis B surface antigen positive or core antibody positive, hepatitis C patients, continuous antiviral therapy is recommended to prevent viral activation.
5. Patients with renal failure requiring hemodialysis or peritoneal dialysis;
6. History of immunodeficiency, including HIV positive or suffering from other acquired, congenital immunodeficiency diseases, or history of organ transplantation;
7. Poorly controlled diabetes (fasting blood glucose (FBG) > 10 mmol/L);
8. Urinalysis showed urine protein ≥ + +, and confirmed 24-hour urine protein > 1.0 g;
9. Patients with epilepsy requiring treatment;

2) Tumor related symptoms and treatment:

a) Patients who previously received surgery, chemotherapy, radiotherapy or other anti-cancer therapy 4 weeks before the start of study treatment (the washout

period will be calculated from the end time of treatment); patients who previously received local radiotherapy can be eligible if: the end of radiotherapy is more than 4 weeks from the start of study treatment (more than 2 weeks for brain radiotherapy); and the target lesion selected for this study is not in the radiotherapy area; or the target lesion is located in the radiotherapy area, but progression has been confirmed.

b) Patients who have received Chinese patent medicines with anti-tumor indications (including compound cantharidin capsules, Kangai injection, Kanglaite capsules/injection, Aidi injection, Brucea javanica oil injection/capsules, Xiaoer Fangye/injection, cinobufotalin capsules, etc.) in the package insert of NMPA approved drugs 2 weeks before the start of study treatment;

c) Uncontrolled pleural effusion, pericardial effusion or ascites requiring repeated drainage (judged by the investigator);

d) Patients with brain metastasis whose symptom control is stable for less than 4 weeks after stopping dehydrating agents and steroid drugs;

3) Study Treatment Related:

a) History of live attenuated vaccination 28 days before the start of study treatment or planned live attenuated vaccination during the study;

b) Previous history of severe allergy to macromolecular drugs, or allergy to known components of TQB2858 injection;

c) Active autoimmune disease requiring systemic therapy (eg, use of disease-modifying drugs, corticosteroids, or immunosuppressive agents) within 2 years of the start of study treatment. Replacement therapy (e.g., thyroxine, insulin, or physiological corticosteroids for adrenal or pituitary insufficiency, etc.) is not considered systemic;

d) Diagnosis of immunodeficiency or ongoing systemic glucocorticoid therapy or any other form of immunosuppressive therapy. (Dose > 10 mg/day prednisone or other effective hormones), and the drug is still in use 2 weeks before the first dose;

4) 4. Participated in other clinical trials of anti-tumor drugs;

5) Patients with concomitant diseases that, in the judgment of the investigator, seriously endanger the safety of the subjects or affect the completion of the study, or are considered unsuitable for the subjects for other reasons;

4.3 Study Exit or Termination

4.3.1 Criteria for Study Withdrawal

Subjects may withdraw from the trial at any time on their own, or be withdrawn by the Investigator for safety or behavioral reasons, or because they are unable to comply with protocol-required study visit times or procedures at their site.

Reasons for withdrawal from the study may include:

1. Subject withdraws consent;

Note: Withdrawal of consent by default means that the patient can no longer be followed; the subject may also choose to discontinue study treatment.

2. The situation that the investigator considers necessary to withdraw from the study;
3. Lost to follow-up;
4. The subject died.

4.3.2 Criteria for Discontinuation of Study Treatment

Discontinuation of trial treatment does not constitute withdrawal from the study. Subjects who discontinue trial treatment must continue to complete the remaining visits as required by the protocol. The study treatment must be discontinued if the subject meets any of the following criteria:

1. The subject requests to discontinue the investigational drug;
2. The efficacy evaluation meets the criteria for disease progression confirmed by the investigator (according to RECIST 1.1 criteria);
3. The subject experiences a pregnancy event during the course of the study;
4. The subject is still unable to tolerate the toxicity after dose adjustment, or develops adverse events, laboratory abnormalities or concurrent diseases,

etc., and continues to participate in the study is not in the best interest of the subject as judged by the investigator;

5. Worsening of clinical symptoms (e.g. ECOG score ≥ 2 Or ≥ Grade 2 dyspnea caused by persistent increased pleural effusion, etc.), making it impossible to continue the trial;
6. Significant protocol deviations such as subject ineligibility are found after enrollment;
7. Subjects who, in the opinion of the investigator, are noncompliant with study procedures or study drug administration should consult with the sponsor for instruction on handling the subject;
8. Other reasons that, in the opinion of the investigator, make continuation of study treatment impossible.

4.3.3 Discontinuation from Study or Study Treatment of Steps

If a subject was withdrawn from the study or discontinued study treatment, the following actions were taken:

1. The reason (s) must be documented in the medical file and eCRF;
2. An End of Treatment (EOT) visit should be performed. Tests done during the window before treatment withdrawal do not need to be done at the EOT visit Inspections completed above, refer to the trial visit schedule;
3. The subject should be followed for safety for 30 days after the EOT visit or the subject starts a new anticancer therapy;
4. Subjects without disease progression should continue to undergo imaging evaluation at the scheduled frequency and time until the patient starts new anti-tumor treatment or disease progression. Every effort should be made to obtain imaging evidence of disease progression in such subjects;

4.4 Lost to Follow-Up

If a subject fails to return for a scheduled visit and the site staff is unable to contact him/her for at least 3 times, he/she will be considered lost to follow-up.

Contact information includes telephone, fax, SMS, social media tools and mail,

etc. All such attempts to contact the patient should be documented in the subject's medical record or in the study file. If it is determined that a subject has died, the study site will make every effort to obtain death information and cause of death, such as using public resources, such as community health registries and databases. If the subject's status is not available despite all attempts, the investigator should report the date the subject was last known to be alive and record in the subject's medical history.

4.5 Exclusion criteria

Subjects will be excluded if they meet any of the following criteria:

1） Patients who do not meet the inclusion and exclusion criteria are included in this study by mistake;

2） Wrong dose and method of administration;

3） Use of other chemotherapy, surgical treatment or investigational drug other than those specified in this protocol during the trial;

4） A serious deviation or violation of the protocol that affects the evaluation of the safety or efficacy of the investigational product.

The final eliminated cases shall be subject to the division decision of statistical analysis data set.

4.7 Criteria for Continuing Treatment After Disease Progression

Subjects may continue to receive study treatment if, in the opinion of the investigator, there is still a clinical benefit to the subject from receiving study treatment and the potential benefits of continuing study treatment outweigh the risks.

The investigator should record in detail the reasons for subjects to continue treatment, and subsequent observation and evaluation should still be performed according to the procedures specified in the clinical trial.

4.8 Premature termination or suspension of the trial

The sponsor, the ethics committee, and the investigator have the right to terminate this study at any time. Reasons for early termination or suspension include but are not limited to:

1) The number and severity of adverse events suggest that it will bring significant

harm to the subjects;

2) Administrative department cancels;

3) Spontaneous by the sponsor.

5. Treatment of Subjects

5.1 Basic information of investigational drug

Name: TQB2858 Injection

General Properties: Colorless to pale yellow, clear, sterile solution for injection, preservative-free

Manufactured by: Chia Tai Tianqing Pharmaceutical Group Co., Ltd ./Chia Tai Tianqing Pharmaceutical Group Nanjing Shunxin Pharmaceutical Co., Ltd.

Strength: 200 mg (4 ml)/vial

Packaging: 10 bottles/box;

Storage condition: Store at 2 ~ 8°C, protected from light;

Batch number: subject to the CoA used during the trial.

Validity period: subject to the CoA used during the trial.

5.2 Packaging of Investigational Product And label

The package should be printed with the information of investigational drug name, drug batch number, strength, storage conditions, shelf life, manufacturer name, etc., and marked with "for clinical study only".

5.3 Storage and management of investigational drug Process

5.3.1 Drug Receipt

After the investigational drug arrives at the study site, the authorized drug administrator receives the investigational drug, verifies whether the temperature records in the process of drug transportation meet the requirements, verifies the transportation sheet, drug package, drug quantity, drug batch number and drug shelf life, and simultaneously registers the drug for warehousing. The courier floors need to be kept.

5.3.2 Drug storage

At the study site, the authorized Drug Administrator had the responsibility to retain

the study drug and correctly manage and store the study drug. The study drugs must be stored in an appropriate place and documented according to the storage conditions in the certificate of analysis provided by the sponsor. The investigational drug must be stored in a locked cabinet, only the drug administrator and the authorized investigator can get the investigational drug, and the records of receipt, distribution and recovery of the investigational drug must be maintained. At the study site, the drug administrator is responsible for explaining the reasons for any deviation in the storage of investigational drugs (e.g., overheating, loss, etc.) and timely informing the monitor of this project.

5.3.3 Drug distribution Recovery

For the determination of subjects, the investigator should issue an electronic or paper prescription, determine the authorized person to the drug administrator to obtain the drug according to the prescription, and distribute the drug to the relevant personnel. The prescription should be archived for future reference. The drug administrator should timely record the dispensing of each drug in the relevant form. Distribute and recover the drugs according to the cycle. The drug administrator shall recover the remaining drugs and packages, and fill in the drug record related forms. In this study, empty boxes and unused study drugs were returned to the sponsor after being counted by the monitor at regular intervals or at the end of the trial.

5.4 Investigational Drug usage and dose adjustment

5.4.1 Method of Administration

Administration dose: RP2D dose determined in phase 1.

Administration method:

TQB2858 injection: once every 3 weeks (dosing window: ± 3 days), 3 weeks as a treatment cycle, until disease progression or investigator's judgment is not appropriate to continue the treatment. According to the determined RP2D dose, the given dose of TQB2858 was diluted with 0.9% sodium chloride injection [0.9% (w/v) sodium chloride solution, the same below] to a final protein concentration of 0.1 mg/ml ~ 10 mg/ml (such as 30-250 ml), and the entire contents of the intravenous infusion bag were intravenously infused into the body using an infusion set with an inline filter of 0.2 µm

or 0.22 μm, with an infusion time of 20-60 ± 10 min. The infusion time starts from the start of infusion of TQB2858, ends at the end of infusion of TQB2858 and the end of flushing with 0.9% sodium chloride injection (it is recommended to flush with 20ml 0.9% sodium chloride injection).

In case of infusion reactions, appropriate intervention measures are recommended in "Recommendations for the Management of Immune-Related Adverse Events Caused by Immune Checkpoint Inhibitor Therapy" and "Chinese Society of Clinical Oncology (CSCO) Guidelines for the Management of Immune-Checkpoint Inhibitor-Related Toxicity 2019".

Preparation method: Under aseptic operation, professionals discard a certain volume of 0.9% sodium chloride injection from the infusion bag with 250 ml package size of 0.9% sodium chloride injection. Discard an equal volume of 0.9% Sodium Chloride Injection, TQB2858 Injection, to the infusion bag. Gently invert the bag without shaking to ensure that the solution is well mixed. After mixing well, the prepared solution is clear by visual inspection, without turbidity, precipitation or other quality problems, and then can be used.

Notes: This product is Sterile, clear, colorless to slightly yellow, preservative-free solution for injection. Carefully inspect each vial of TQB2858 injection before use to confirm that it is not damaged and that the solution in the vial is not discolored, coagulated, turbid, or precipitated before use. Because this product contains no preservatives and can only be used once, the remaining solution in the opened bottle after use should be discarded or disposed of according to local requirements.

Do not mix different batches of drug product; ensure that the prepared TQB2858 infusion solution is clear or clear and does not appear Quality problems such as turbidity and precipitation;

It is recommended that the prepared TQB2858 infusion be used immediately after preparation. If the prepared TQB2858 infusion solution cannot be used immediately, it should be stored at 2 ~ 8°C for no more than 24 hours, or at 25°C below for no more

than 4 hours (storage time: the time immediately after opening the TQB2858 injection vial to the start of injection use).

Avoid infusion of TQB2858 in TQB2858 mixed with other drugs and avoid intravenous bolus injection of TQB2858.

Subjects who, in the opinion of the investigator, are benefiting from the drug and who agree to continue the drug will be provided with the study drug until disease progression or the investigator judges that the subject is no longer suitable for the drug.

5.4.2 Medication Modifications

1. Provisions on medication adjustment of TQB2858:

No dose adjustment is allowed.

Delayed or discontinued medication

TQB2858 injection was allowed to be delayed. If TQB2858 injection caused adverse events resulting in delayed administration and the administration could not be resumed after more than 12 weeks, the treatment with TQB2858 injection should be permanently discontinued.

2. Suggestions on the handling of adverse drug events

Refer to Treatment Recommendations for Immune-related Adverse Events Caused by Immune Checkpoint Inhibitor Therapy and Chinese Society of Clinical Oncology (CSCO) Guidelines for the Management of Immune-Checkpoint Inhibitor-associated Toxicity 2019 for the treatment recommendations for adverse events caused by TQB2858 injection.

5.5 Concomitant Medication and Concomitant Therapy

5.5.1 Prohibited Concomitant Medication or Treatment

Subjects may receive other medications that, in the opinion of the investigator, are medically necessary, but not unequivocally required by the non-protocol for systemic treatment of cancer of medication or treatment, such as chemotherapy, radiotherapy, immunotherapy, anti-tumor biological therapy, anti-cancer Chinese patent medicines or Chinese herbal medicines, etc., and cannot participate in clinical studies of cancer

treatment.

Subjects must not be vaccinated against live or infectious diseases, including measles, mumps, rubella, varicella, yellow fever, seasonal influenza, H1N1 influenza, rabies, BCG, and typhoid fever, after the first dose of study treatment and within 60 days after last dosing.

5.5.2 Concomitant Medications or Treatments Used with Caution

TQB2858 injection is an anti-PD-L1/TGF-β bifunctional fusion protein that belongs to cancer immunotherapeutic drugs, so it is not recommended to use hormones and/or drugs or treatments with immunoregulatory functions, so as not to affect the efficacy of TQB2858. These agents or treatments may be selected with caution if deemed necessary by the investigator (e.g., topical steroids for rash relief).

5.5.3 Permitted Concomitant Medications or Treatment

Non-conventional therapies (eg, herbal remedies or acupuncture) and vitamin/mineral supplements may be used if there is no effect on the endpoints of the study in the opinion of the investigator.

Patients may receive bisphosphonates for treatment of bone metastases during treatment. Palliative small-area radiation therapy is permitted if painful bone metastases cannot be effectively controlled by systemic therapy or local analgesia.

To prevent infusion reactions, chemoprophylaxis may be considered 30 to 60 minutes before each infusion of TQB2858, and H1 blockers (diphenhydramine or equivalent) and acetaminophen are recommended. Systemic corticosteroid therapy required to control infusion reactions or immune-related adverse events must be maintained at the tapering dose for at least 4 weeks prior to the next dose of TQB2858, and the therapeutic dose does not suppress immune system function (prednisone ≤ 10 mg/day, or equivalent ≤ "prednisone 10 mg equivalent dose").

5.5.4 Start and End Time Points for Collection of Concomitant Medications and Treatments

In the course of trial observation, all the concomitant medications other than the

study drug should be recorded in the subject's medical record and EDC. The recording time and requirements of concomitant medication in this trial are as follows:

Time period	Record Requirements
From signing of ICF to before first dose of study drug	All medications and significant non-drug therapies were recorded, including: generic drug name and daily dose; reason for drug therapy; start and end dates of drug therapy or whether drug was continued at study entry.
From the start of the first dose to withdrawal from the trial	All drug treatments and significant non-drug treatments were recorded.
Withdrawn from the trial until 28 days after initiation of study drug or initiation of other antineoplastic therapy (whichever occurred first)	Only all medications corresponding to the disposition of the AE will be recorded.
Post-treatment period (after administration of 28 mg or initiation of other antineoplastic therapy until end of trial)	Only anti-tumor related therapies will be recorded (only recorded in the Post-OS Follow-up section).

6. Test Flow

6.1 Screening Visit

The screening period is from 28 days before the first dose to 1 day before the first dose, recorded as D-28 ~ D-1. Observations and assessments need to be completed during the screening period for:

1) Signed informed consent (completed prior to items 2-12 below and prior imaging studies may be completed prior to signing informed consent);
3) Pathological reports, disease stages, and molecular characteristics of the disease were collected for the diagnosis of advanced high-grade sarcoma. D-28 to D-1 done;
4) Tissue sections or biopsies were collected for PD-L1, TGF-β and other expression detection (for details, see Fig. 7.4 Section). D-28 to D-1 done;
5) Demographic data: date of birth, age, gender, ethnicity, occupation. D-28 to D-1 done;
6) Medical history Treatment history (completed on D-28 ~ D-1): previous treatment regimen (surgery, chemotherapy, radiotherapy, targeted therapy, other anti-tumor therapy), previous major diseases, concomitant diseases and treatment allergy history, smoking history;
7) Concomitant medications and adverse events were recorded. D-28 to D-1 done;

8) Imaging examination (completed on D-28 ~ D-1):

- Contrast-enhanced CT/MRI (the examination sites include: neck, chest, abdomen, pelvic cavity or other differential examinations which may include the above areas, as well as the suspected lesion sites);
- Contrast-enhanced MRI is recommended for head examination. If MRI cannot be performed due to contrast agent allergy or other reasons, contrast-enhanced CT is feasible;
- Patients with bone metastasis or symptoms of bone metastasis should receive bone scan;
- If imaging has been performed within 28 months (within 3 months for bone scan) before the first dose, and the same method is used in the same center, it can be used as the baseline imaging index.

9) Overall response (D-7 ~ D-1 completed, the subsequent visits of specific items are the same and not listed in detail any more):

- Height, weight, ECOG score;
- Vital signs: body temperature, pulse, respiration and blood pressure;
- Physical examination: examination of various tissues and organs (skin, mucosa, lymph nodes, head, neck, chest, abdomen, spine/limbs, nervous system, etc.);

10) 12-Lead ECG, echocardiography: Echocardiography should include score (LVEF). Completion of D-7 ~ D-1;

11) Blood pregnancy (completed on D-7 ~ D-1, examined in female subjects of childbearing potential): human chorionic gonadotropin (HCG) level will be measured;

12) Infection screening (completed on D-7 ~ D-1): hepatitis B surface antigen (HbsAg) (hepatitis B virus deoxyribonucleic acid HBV-DNA should be additionally measured if HBsAg or HBeAg is positive), hepatitis B surface antibody (HBsAb), hepatitis B e antigen (HBeAg), hepatitis B e antibody (HBeAb), hepatitis B core antibody (HBcAb), hepatitis C antibody (Anti-HCV) (hepatitis C virus ribonucleic

acid HCV-RNA should be additionally measured if hepatitis C antibody is positive), and human immunodeficiency virus antibody (Anti-HIV);

13) Laboratory tests (completed on D-7 ~ D-1. See the table below for specific items. The subsequent visits are the same and will not be listed in detail);

Table 5 List of specific laboratory test items

Inspection category	Inspection item
Blood routine	White blood cell count (WBC), neutrophil count (NEUT), red blood cell count (RBC), hemoglobin (HGB), hematocrit (HCT), platelet count (PLT), lymphocyte count (LYM), monocyte count (MONO), eosinophil count (EOS), basophil count (BASO)
Urine routine	Urine red blood cell (RBC-U), urine white blood cell (WBC-U), urine protein (PRO-U) (add 24-hour urine protein quantification if necessary), urine glucose (GLU-U), urine pH (PH) and urine occult blood (BLD)
Stool routine	Fecal white blood cells (WBC), fecal red blood cells (RBC), fecal occult blood (OCCBLD)
Blood biochemistry	Liver function: total bilirubin (TBIL), direct bilirubin (DBIL), alanine aminotransferase (ALT), aspartate aminotransferase (AST), alkaline phosphatase (ALP), serum albumin (ALB), serum globulin (GLOB), serum total protein (TP), γ-glutamyltransferase (GGT), lactate dehydrogenase (LDH);<br>Renal function: blood urea nitrogen (BUN)/urea (UREA), creatinine (CRE); fasting blood glucose (GLU);<br>Electrolytes: potassium (K), sodium (Na), chloride (Cl), calcium (Ca), magnesium (Mg), inorganic phosphorus (PHOS);<br>Four lipid items: total cholesterol (TC), triglyceride (TG), high-density lipoprotein (HDL), low-density lipoprotein (LDL), and uric acid (UA)
Coagulation function	International normalized ratio (INR), prothrombin time (PT), activated partial thromboplastin time (APTT), thrombin time (TT), fibrinogen (Fbg), D-dimer
Thyroid function	Free triiodothyronine (FT3), free thyroxine (FT4) and thyroid-stimulating hormone (TSH) (if conditions permit, additional tests of triiodothyronine T3 and thyroxine T4 are required)
Cardiac function	Creatine kinase (CK), creatine kinase isoenzyme (CK-MB), cardiac troponin I (cTnI), cardiac troponin T (cTnT), brain natriuretic peptide (BNP) and/or N-terminal pro-brain natriuretic peptide (NT-proBNP)

6.2 TREATMENT PERIOD VISITS

The safety visit and efficacy visit during the treatment period are the treatment period visits to monitor the effectiveness and safety of the subjects during the treatment

period.

If the subject's medication is delayed for any reason, the time point for safety visit shall be postponed, but the time point for efficacy visit shall not be postponed.

6.2.1 Cycle 1

Cycle 1 consists of 21 days, which is recorded as C1D1 ~ C1D21. Observations and assessments need to be completed during this period for:

1) C1D1 visit

- TQB2858 administration;
- Vital signs and body weight;
- Record concomitant medication and adverse event;

2) C1D7 visit

- Comprehensive: body weight, vital signs, physical examination;
- Laboratory tests: blood routine, blood biochemistry;
- 12-ECG: if chest pain, palpitations and other symptoms occur, echocardiography, myocardial enzyme spectrum and troponin should be additionally examined, the same below;
- Record concomitant medication and adverse event;

3) C1D14 visit

- Comprehensive: body weight, vital signs, physical examination;
- Laboratory tests: blood routine, blood biochemistry;
- 12-ECG;
- Record concomitant medication and adverse event;

4) C1D21 visit (± 3 days)

- Comprehensive: body weight, vital signs, physical examination;
- Laboratory tests: blood routine, urine routine, stool routine, blood biochemistry, coagulation function, thyroid function and cardiac function;
- 12-ECG;
- Concomitant medications and adverse events were recorded.

6.2.2 Cycle 2

Cycle 2 lasts for 21 days, recorded as C2D1 ~ C2D21. Observations and assessments need to be completed during this period for:

1) C2D1 visit

- TQB2858 administration;
- Vital signs and body weight;
- Concomitant medications and adverse events were recorded.

2) C2D21 visit (± 3 days)

- Comprehensive: body weight, ECOG score, vital signs, physical examination;
- Laboratory tests: blood routine, urine routine, stool routine, blood biochemistry, coagulation function, thyroid function and cardiac function;
- 12-ECG;
- Record concomitant medication and adverse event;
- Imaging (required for subsequent treatment phase visits):
  - ➢ Contrast-enhanced CT/MRI may be performed at the site of target and non-target lesions or other sites that the investigator feels are necessary. The same test method should be used during the screening period;
  - ➢ Contrast-enhanced MRI is recommended for head examination. If MRI cannot be performed due to contrast agent allergy or other reasons, contrast-enhanced CT is feasible; if there is no brain metastasis lesion during the screening period, head imaging examination should be performed when there are symptoms of brain metastasis or the investigator considers necessary;
  - ➢ Bone scan should be performed for patients with bone metastasis or symptoms of bone metastasis; if there is no bone metastasis during the screening period, bone scan should be performed when there are symptoms of bone metastasis or the investigator considers necessary;
  - ➢ Allowed to be completed within 7 days (± 7 days) before and after this

visit;

6.2.3 Cycle 3 and subsequent singular cycles

1) C (2n + 1) D1 visit

- TQB2858 administration;
- Vital signs and body weight;
- Concomitant medications and adverse events were recorded.

2) C (2n + 1) D21 visit (± 3 days)

- Comprehensive: body weight, vital signs, physical examination;
- Laboratory tests: blood routine, urine routine, stool routine, blood biochemistry, coagulation function, thyroid function and cardiac function;
- 12-ECG;
- Concomitant medications and adverse events were recorded.

6.2.4 Cycle 4 and Subsequent Even Cycles

Imaging visits: visits will be performed every 2 cycles within 1 year after the start of the study; visits will be performed every 4 cycles if the study duration exceeds 1 year (except for pseudoprogression confirmation and confirmation of PR and CR) until subject's progression out or the end of the study.

1) C (2n + 2) D1 visit

- TQB2858 administration;
- Vital signs and body weight;
- Record concomitant medication and adverse event;

2) C (2n + 2) D21 visit (± 3 days)

- Comprehensive: body weight, ECOG score, vital signs, physical examination;
- Laboratory tests: blood routine, urine routine, stool routine, blood biochemistry, coagulation function, thyroid function and cardiac function;
- 12-ECG;
- Record concomitant medication and adverse event;
- Imaging examination;

6.2.5 End of Treatment (EOT) Visit

When a subject meet any of the reasons for the end of study treatment, relevant examination procedures for the end of treatment (EOT) visit are required. These tests do not have to be performed at the EOT visit if some of them were done within 7 days before treatment withdrawal. The following tests/procedures should be completed or the following information should be collected at the EOT visit (within 7 days after the end of treatment):

- Comprehensive: body weight, ECOG score, vital signs, physical examination;
- Laboratory tests: blood routine, urine routine, stool routine, blood biochemistry, coagulation function, thyroid function and cardiac function;
- 12-ECG;
- Recording concomitant medication, adverse events and drug recovery;
- Imaging examination (this examination is not required if there is corresponding imaging record due to disease progression);

6.2.6 Follow-up Visit

After the end of treatment visit, enter the follow-up period. The safety follow-up period is the interval between the EOT visit and the scheduled follow-up visit, which should occur 30 days (± 7 days) after the EOT visit or 30 days (± 7 days) after study drug administration if the EOT visit was not performed. Adverse events and SAEs must be reported to at least 30 days after initiation of study drug, the date of the follow-up visit, or until the toxicities resolve, return to baseline, or are deemed irreversible, whichever is longer. Reasonable efforts should be made to have the subject return for the follow-up visit and report all AEs that occur during this period.

If a subject is scheduled to start a new antineoplastic therapy before the end of the safety follow-up period, the safety follow-up visit should be performed before the start of the new antineoplastic therapy. Once new antineoplastic therapy was initiated, subjects entered the survival follow-up period.

After completion of the safety follow-up period, enter into the survival follow-up

period. The first survival follow-up period (including telephone follow-up) occurred 8 weeks (± 7 days) after the last safety follow-up and occurred on an 8-week (± 7 days) basis until the subject died, lost to follow-up, the study was terminated by the sponsor, or other study closure criteria were met, whichever occurred first. The investigator may follow up the subject, his/her family or the responsible physician by phone to collect whether other anti-tumor treatment was given to the subject before this visit. If other treatments are used, the treatment regimen and cycle number and outcome must be recorded. The patients were followed up until death, and the relevant cause of death and specific time were recorded to obtain overall survival (OS).

For subjects who discontinue study treatment due to intolerance or other reasons, if no radiographic progression is observed, radiographic assessment should still be performed at the original frequency until disease progression or initiation of other anti-tumor therapy (except anti-tumor therapy with traditional Chinese medicine), and radiographic evidence of disease progression should be obtained as much as possible for such subjects.

6.2.7 Unscheduled Visits

Unscheduled visits may be performed at any time at the discretion of the investigator and appropriate clinical and laboratory tests may be performed based on AEs or other findings.

7. Biomarker study

To explore the expression of CD68, CD163, CD206, IRF8, CD3, CD8, PD-L1, CD20, CD56, B7-H3, CD47 and CD4 in tumor tissues, and to determine the presence of tertiary lymph node structure in tumor tissues based on the positive results in the above tissue sections, 6 white tumor tissue slices (5 microns, for smear and baking gel staining and immunohistochemistry) will be collected from subjects before administration in this study to clarify the characteristics of immunogenic microenvironment of TQB2858 in subjects with advanced high-grade sarcoma.

The collection, numbering, transportation, processing, storage and other detailed

operations of biological samples shall be performed in accordance with the relevant standard operation manual or operating procedures.

8. Efficacy evaluation

TQB2858 injection is a tumor immune drug, and based on the experience of drugs in the same class, the subject may have false progression, i.e. The response assessment criteria for this study were based on RECIST 1.1, while iRECIST criteria were used to confirm response.

Beginning with Cycle 1 Day 1, efficacy will be assessed every 2 cycles over 1 year from the start of the study and every 4 cycles over 1 year until the subject experiences radiologically confirmed disease progression that does not change in frequency due to delay or interruption of treatment. If a subject discontinues study treatment for reasons other than disease progression, tumor imaging assessments at the aforementioned frequency should continue until the subject starts a new antineoplastic therapy, or radiographic evidence of disease progression, or the subject withdraws, or the subject dies, whichever occurs first.

CT or MRI can be used for tumor imaging evaluation at the discretion of the investigator, but the evaluation method, machine and technical parameters should be consistent throughout the study; if there is no contraindication, contrast agent should be used. If imaging has been performed within 28 months (within 3 months for bone scan) before the first dose, and the same method is used in the same center, it can be used as the baseline imaging index. Baseline Imaging examination CT or MRI of the neck, chest, abdomen, and pelvis should be included. Enhanced MRI (recommended) or enhanced CT is required at screening. Imaging should be performed at all suspicious lesion sites. For patients with bone metastasis, bone scan should be used for follow-up of lesions. For patients with bone metastasis, if there is no aggravation of clinical symptoms, each tumor assessment is not required for reexamination. If there is aggravation of clinical symptoms, reexamination should be performed in time. For patients with suspected disease progression before the next scheduled assessment,

unscheduled tumor assessment should be performed. During the trial, only the imaging examination of the lesion site is performed. If there is a suspicious site during the trial, the image of the corresponding site can be additionally examined.

In this study, we preliminarily investigated the therapeutic effect of TQB2858 injection in advanced high-grade sarcoma and assessed the efficacy according to RECIST 1.1 evaluation criteria and iRECIST evaluation criteria. Indicators include objective response rate (ORR), disease control rate (DCR), progression-free survival (PFS), 3-month ORR, overall survival (OS) and duration of response (DOR).

1) Objective response rate (ORR): is the proportion of subjects whose tumor volume reduction reaches the prespecified value and maintains the minimum time limit (at the next imaging assessment), including cases of complete response (CR) and partial response (PR);
2) Disease control rate (DCR): the proportion of patients whose tumors shrink or remain stable for a certain period of time, including patients with CR, PR and stable disease (SD);
3) Progression-free survival (PFS): The time from the first dose to disease progression (PD) or death before PD; if there is no PD or death before PD, the date of the last imaging assessment will be used as the cut-off date;
4) Overall survival (OS): The time from the first treatment of a patient to death from any cause.
5) DOR: In subjects with CR or PR, the time from the first tumor assessment of CR or PR to PD or death before PD; if a subject with CR or PR did not experience PD or death before PD, the date of the last radiographic assessment will be used as the cut-off date.

9. Safety evaluation

9.1 Adverse events

An adverse event (AE) is any untoward medical occurrence in a subject administered an investigational product and which may manifest itself by symptoms,

signs, disease, or laboratory abnormalities and which do not necessarily have a causal relationship with the investigational product. AEs in this trial should be collected from the time the subject signs the informed consent form to 30 days after initiation of treatment or initiation of treatment for a new target indication.

9.2 Evaluation of Adverse Events

The nature and severity of adverse events were evaluated according to the National Cancer Institute Common Terminology Criteria for Adverse Events (NCI-CTCAE v5.0). Items that cannot be assessed by this criterion can be assessed by the following criteria:

Table 6 Unlisted in NCI-CTCAE v5.0 Criteria for Judging the Severity of Adverse Events

CTCAE Level	Degree Level	Definition
Level 1	Mild	Discomfort that does not interfere with daily activities.
Grade 2	Moderate	Discomfort that decreases or interferes with usual activities; there is no indication for treatment or medical intervention, although it may improve the subject's general state of health or symptoms.
Grade 3	Severe	Inability to work and perform usual activities; indications for treatment or medical intervention to improve overall health status or symptoms; delay in starting treatment does not put the patient's survival at immediate risk.
Grade 4	Life-threatening/disabling	Permanent mental or physical condition that is immediately life-threatening or results in disruption of work or normal daily activity; requires medical or medical intervention to maintain survival.
Grade 5	Death	Adverse events may result in death.

9.3 Recording of Adverse Events

During the AE reporting period stipulated in the protocol, the investigator is responsible for collecting all AEs (including SAEs) and recording them in the CRF/eCRF. When recording AEs, the investigator should use correct and standardized medical terminology to avoid colloquialisms and abbreviations. The start time of AE, the highest degree of NCI-CTCAE v5.0 grade, the end time, the correlation with the study drug, the impact on the study, the presence or absence of concomitant treatment and the recovery should be recorded.

Diagnosis vs Symptoms and Signs

If a diagnosis is already present, it should be recorded in the CRF/eCRF rather than individual signs and symptoms (e.g., record liver failure rather than jaundice, elevated transaminases, and throbbing tremor). However, if symptoms and signs cannot be classified as a single diagnosis at the time of reporting, each individual event should be recorded as an AE in the CRF/eCRF. If the diagnosis is later established, it should be updated on the CRF/eCRF to record the diagnosis.

Adverse Events Secondary to Other Events

In general, AEs secondary to other events (e.g., caused by other events or clinical sequelae) should have their primary event recorded, unless the secondary event is more severe or an SAE. However, secondary events with significant clinical significance should be recorded as independent AEs in the CRF/eCRF if they occur at a different time from the primary event; if the relationship between the events is unclear, the primary event and secondary event should be recorded separately.

Continuous, intermittent or isolated adverse events (frequency of adverse events)

Continuous AE refers to an AE persisting during the whole process without remission, for example, upper respiratory tract infection lasting for 5 days. Only one AE should be recorded in the CRF/eCRF. For severity assessment, the most severe severity throughout the event should be recorded.

Intermittent AEs are AEs that change or are relieved in symptoms, signs or laboratory parameters during the whole process, but do not have clinically significant outcomes, such as nausea and vomiting that last for many days, during which there is relative remission; subjects with hypertension have a relatively continuous course of hypertension despite intermittent remission in multiple blood pressure tests. Such AEs may be recorded in only one CRF/eCRF. The most severe severity over the course of the event should be recorded during the intensity assessment.

A separate AE (Single AE) is defined as an AE that can logically only occur alone, or that occurs only once independently in a trial, for example, an accident in which a

subject falls while on medication; and vomiting that a subject only experiences once during a trial. Only one such AE should be recorded on the CRF/eCRF.

It should be noted that if the above AE has been significantly clinically significant recovered, but the same AE occurs later and the latter is considered to have no continuous course with the former, the occurrence of the event should be recorded separately in the CRF/eCRF.

Laboratory abnormalities or vital signs

All laboratory test results may be recorded on the laboratory results page of the CRF. Not all laboratory abnormalities/vital sign abnormalities should be recorded as AEs, and it is the responsibility of the investigator to review all laboratory abnormalities and abnormal vital signs and make medical judgment as to whether they should be recorded as AEs. Any of the above abnormalities with significant clinical significance, for example, one or more of the following conditions should be recorded as AE:

- Accompanying clinical symptoms
- Results in a change in study medication (e.g., dose modification, temporary or permanent discontinuation)
- Requires medical intervention or change in concomitant therapy (e.g., increase, suspension, discontinuation, or other change in concomitant medication, therapy, or treatment)
- Clinically significant as judged by the investigator

If a clinically significant laboratory abnormality or vital sign abnormality is a sign of a disease or syndrome (e.g., elevated ALT/AST and blood bilirubin due to hepatic impairment), only the diagnosis (i.e., hepatic impairment) should be recorded on the Adverse Event form of the CRF/eCRF. Otherwise, the laboratory test abnormality or vital sign abnormality should be recorded in the adverse event form of CRF/eCRF, indicating whether the test value is higher or lower than the normal range. If there are standard clinical terms corresponding to the laboratory abnormalities or vital sign abnormalities, the clinical term should be recorded in the CRF/eCRF

(e.g.,"hyperkalemia"should be recorded when blood potassium increases to 7.0 mmol/L).

Death

When recording a death event, if there is an AE leading to death, the AE leading to death should be recorded in the CRF/eCRF using a single medical concept and the event should be reported as an SAE; if the cause of death is unknown, "unexplained death" should be recorded in the AE form of the CRF/eCRF and reported as an SAE first, followed by further investigation into the exact cause of death, and the record and SAE report should be updated after the cause of death is known; if the death is due to tumor progression, it should not be recorded and reported as an AE/SAE.

Pre-existing medical conditions

The preexisting symptoms/signs a subject has already presented during the screening period of the trial will be recorded as AEs only when there is worsening in severity, frequency, or nature (except for worsening of the medical condition under study) after entry into the trial. Changes from the previous state should be reflected in the records, such as "increased frequency of headache", "aggravated hypertension", etc.

Hospitalization, prolonged hospitalization

The following situations result in hospitalization or prolonged hospitalization, which should not be reported as SAE:

Planned hospitalization or prolongation of hospitalization as required by the protocol (e.g., for dosing, efficacy assessment, etc.); Hospitalization for a medical condition that was present and unchanged prior to study participation, such as an elective procedure or treatment that was scheduled prior to study participation, the subject was hospitalized as scheduled for the procedure or treatment that occurred after study participation, is not considered an adverse event. However, if the condition of the existing disease worsens during the study (e.g., surgery or treatment should be performed earlier than originally planned), hospitalization for surgery or treatment is required due to the deterioration of the disease, and the deterioration of the condition

will be considered as an SAE.

Surgery

If the condition being treated by the procedure is known, the condition should be recorded as the AE rather than the procedure itself (e.g., if the subject has undergone inguinal hernia repair, an"inguinal hernia"should be recorded instead of"inguinal hernia repair"); however, if the reason for the procedure is unclear, the procedure may be recorded as the AE (e.g., if the subject has undergone abdominal exploration, an"abdominal exploration"may be recorded as the AE).

Pregnancy

If a female patient or a female partner of a male patient becomes pregnant during the clinical trial, the investigator should be notified immediately. The investigator should fill out the "Serious Adverse Event Report Form" within 24 hours after learning of the pregnancy event and report to the sponsor and the Ethics Committee, and follow up should continue until the outcome of pregnancy (such as termination of pregnancy, delivery), and report the results to the sponsor and the Ethics Committee. If a female patient becomes pregnant, study drug should be discontinued immediately and the investigator should discuss with the patient the risks of continuing the pregnancy and the possible effects on the fetus.

Artificial/spontaneous abortion, termination of pregnancy for medical reasons or fetus/newborn congenital abnormality or malformation during pregnancy should be considered as SAE, which should be recorded and reported according to the time limit for SAE.

Disease progression

It should not be considered as AE if the occurrence of an event was clearly consistent with the expected pattern of progression of the primary tumor. Hospitalization solely due to progression of this disease is also not considered an SAE. If symptoms cannot be confirmed to be completely caused by disease progression, or it is inconsistent with the expected pattern of tumor progression, relevant clinical

symptoms may be recorded as AE, which conforms to SAE reportable SAE.

9.4 Follow-up of Adverse Events

The investigator should follow all AEs until occurrence of any of the following situations,

- AE is relieved or improved to baseline level;
- Investigator confirms no further improvement is expected;
- Subject died;
- Subject lost to follow-up;
- The investigator confirmed that the AE was not related to the study treatment;
- Subject starts a new anticancer therapy;
- No clinical or safety data will be collected, or the database will be finally closed.

The final outcome of each AE, including date of AE resolution or death, must be recorded on the CRF/eCRF.

9.5 Criteria for judging the correlation between drugs and adverse events

The investigator should assess the possible association between adverse events and the study drug. See the following 5 criteria for judgment.

(1) Whether there is a reasonable temporal sequence between medication and the occurrence of adverse reactions;

(2) Whether the reaction conforms to the known type of adverse reaction of the drug;

(3) The reaction is relieved or disappeared after drug withdrawal or dose reduction;

(4) Whether the same reaction occurs again after re-administration of the suspected drug;

(5) Whether the reaction can be explained by the effect of concomitant drugs, progression of the patient's condition or other treatment measures;

Table 7 Form for Judging the Relationship between Adverse Event and Drug

	1	2	3	4	5
Definitely related	+	+	+	+	-
Probably related	+	+	+	?	-

Possibly related	+	-	±?	?	±?
Unlikely related	-	-	±?	?	±?
Definitely unrelated	-	-	-	-	-

Note: + indicates yes, — indicates no, ± indicates difficult to confirm or deny, ? indicates that the situation is unknown

The three items judged as definitely related, probably related, and possibly related to the results were counted as adverse reactions, and the incidence of adverse reactions was calculated accordingly.

9.6 Serious Adverse Events

9.6.1 Definition of Serious Adverse Events

An adverse event is classified as serious when the subject experiences an adverse event that meets one or more of the following criteria after receiving the investigational product: death, is life-threatening, results in persistent or significant disability or incapacity, requires or prolongs patient hospitalisation, is a congenital anomaly or birth defect, follows a pregnancy (spontaneous or induced abortion, termination of pregnancy for medical reasons), and is an important medical event (not immediately life-threatening or results in death, but may jeopardise the subject or may require intervention to prevent one of the other outcomes listed above).

Disease progression, including signs and symptoms of progression, should not be reported as a serious adverse event, but death due to disease progression should be reported as a serious adverse event if it occurs during the trial or safety reporting period. During the trial or safety reporting period, if the final outcome of cancer is death, the event that led to death must be reported as a serious adverse event.

9.6.2 Handling of Serious Adverse Events

For any serious adverse event occurred during the clinical trial, the investigator should report to the designated mailbox of PV Department of the sponsor in written form within 24 hours after being informed (TQB2858@cttq.com), followed by detailed

and written follow-up reports in a timely manner. For the reporting of death events, the investigator should provide the sponsor and the Ethics Committee with other required data, such as autopsy report and final medical report. Upon receipt of a serious adverse event, the sponsor shall analyze and evaluate it immediately, including seriousness, correlation with the investigational drug and whether it is an expected event. For suspected and unexpected serious adverse reactions, the sponsor should report them to all investigators participating in the clinical trial of investigational drug, clinical trial institutions and ethics committee in an expedited manner; and the investigator should report suspected and unexpected serious adverse reactions provided by the sponsor to the ethics committee. Suspected and unexpected serious adverse reactions should also be reported by the sponsor to drug regulatory authorities and health authorities.

9.7 Common Adverse Events Disposal measures

Refer to Treatment Recommendations for Immune-related Adverse Events Caused by Immune Checkpoint Inhibitor Therapy and Chinese Society of Clinical Oncology (CSCO) Guidelines for the Management of Immune-Checkpoint Inhibitor-associated Toxicity 2019 for the treatment recommendations for adverse events caused by TQB2858 injection.

10. Data Management

See Data Management Plan and standard operating procedures of data management department for details.

10.1 Data Entry

An electronic data capture system (EDC) was used for data collection and management in this trial. The investigator or clinical coordinator should timely, accurately, completely and normatively enter the source data information into EDC according to the 《eCRF Completion Guidelines》. Only the investigator or clinical coordinator trained in EDC operation is authorized to perform data entry.

10.2 Data Verification and Review

The clinical research associate (CRA) should timely check the EDC data against

the original medical documents to ensure the data are accurate, complete, consistent and standardized. Clinical Data Administrator shall review EDC data according to the Data Verification Plan to ensure the data logic, completeness and standardization.

10.3 Data Cleaning

Data cleaning methods include edit check of EDC system, SAS program verification and manual verification. CRA or Clinical Data Administrator raised a query on the trial data found in EDC in a timely manner, and the Investigator or Clinical Coordinator answered the query or corrected the data in a timely manner. If the query was resolved, the query was closed. If the query still existed, it was required to raise a query for further confirmation, until the query was finally resolved.

10.4 Electronic Signatures

After data cleaning, the investigator finally confirmed the authenticity and accuracy of the entered data, and electronically signed the data in EDC. The investigator who performed the electronic signature was required to sign an Electronic Signature Statement prior to implementation stating that the electronic signature had the same legal effect as a written, handwritten signature.

10.5 Database Lock

All the trial data are entered, data verification, data review and query resolution are completed, electronic signature is completed, medical verification is completed, and after the data review report is finalized, a data review meeting is held to jointly confirm the relevant matters before locking. After the Clinical Data Manager confirms the completion of database lock list, perform database lock. In principle, the database will not be unlocked after locking.

10.6 Data Transfer

After the database is locked, the Clinical Data Manager exports the data for submission to the Statistical Analyst and exports the subject eCRFs to each site for archiving.

11. Statistical Analysis

11.1 Analysis Populations

Full Analysis Set (FAS): All the subjects who were enrolled and took the study drug at least once were included in the FAS. The following situations may lead to the exclusion of the enrolled subjects from the FAS: they did not meet the main criteria; they did not take the study drug once; they did not have any data after enrollment. Full analysis set is used for analysis of baseline and demographic data;

Safety Analysis Set (SS): all subjects who used the study drug at least once and had safety evaluation data; the safety population will be used for the analysis of safety data.

Effective Analysis Set (EAS): all enrolled subjects who have received at least one dose of the study drug and have at least one efficacy evaluation indicator. The purpose of this data set is to analyze the efficacy indicators of subjects.

11.2 Sample Size

The primary objective of this study is to evaluate the efficacy and safety of TQB2858 injection in advanced high-grade sarcoma, and it is planned to include 30 ~ 100 subjects (Cohort 1:10 ~ 30 subjects with primary alveolar soft part sarcoma; Cohort 2:10 ~ 30 subjects with alveolar soft part sarcoma failing to receive PD-1 therapy; Cohort 3:10 ~ 40 subjects with other subtypes (pleomorphic sarcoma, classical osteosarcoma, Ewing sarcoma, chondrosarcoma and dedifferentiated liposarcoma, etc.)). The final sample size is adjusted according to the specific situation of the trial.

11.3 Statistical Analysis

11.3.1 General Principles

PK parameter calculations will be performed using Phoenix WinNonlin 7.0 or later version, and all other statistical analyses will be programmed and calculated using SAS 9.4 or later version of Statistical Analysis Software. Number of cases and missing cases for summary analysis. Quantitative data are generally described by mean, standard deviation, median, quartiles, minimum and maximum. For some quantitative data, additional statistics such as geometric mean and coefficient of variation are analyzed.

Qualitative and hierarchical data are generally described by frequency and percentage. Statistical test showed that the test level was $\alpha = 0.05$, P value ≤ 0.05, which was judged as statistically significant.

11.3.2 Hypothesis Testing

None

11.3.3 Subject Disposition

The exact number of all subjects who entered the study from the screening period to the completion of the study and the number of subjects who were enrolled and included in each analysis set, and the number of subjects who completed the trial and withdrew from the trial was described. Subjects who fail screening will be grouped and summarized by primary reason, and subjects who prematurely discontinue treatment after enrollment will be grouped and summarized by dose group and primary reason.

Subject disposition in each data set: subject disposition in each analysis population was summarized, and the reasons for excluding subjects in each analysis population were summarized. Subjects excluded from each analysis population were tabulated by treatment group (including protocol deviations and reasons for exclusion).

11.3.4 Demographic and Baseline Characteristics

Baseline characteristics were defined as nonmissing assessments before first drug intake.

The demographic characteristics and baseline characteristics of all subjects will be statistically described. For quantitative data, describe the mean, standard deviation, median, quartile, maximum and minimum; for qualitative data, list the frequency and percentage.

11.3.5 Safety Analysis

1) Summarize the exposure of subjects to the study drug, including the number of cycles completed, dose modifications during treatment, and the cumulative number of dose modifications during treatment.

The treatment time, total dose and average daily dose of the investigational drug

during the treatment period will be statistically described.

2) Adverse events

All adverse events (AEs) will be classified according to the current version of the Medical Dictionary for Regulatory Activities (MedDRA) at the time of coding and graded according to CTCAE v5.0.

Treatment Emergent Adverse Events (TEAE)

A TEAE was defined as an adverse event that appeared after or worsened after study drug administration.

a) The following adverse events were summarized by actual treatment:

- All adverse events (AEs);
- Treatment-emergent adverse events (TEAEs);
- TEAE judged to be DLT
- Grade 3 and above TEAEs;
- Serious adverse events (SAEs);
- TEAE leading to dose interruption and termination;
- TEAEs related to the study drug;
- Grade 3 and above TEAEs related to the study drug;
- SAEs related to the study drug;
- TEAEs related to the study drug leading to dose interruption and termination;

b) Describe the number and incidence of adverse events listed above by dose group, sorted by system organ class SOC and preferred term PT.

c) Number of cases and incidence of each toxicity grade of NCI CTCAE version 5.0 will be calculated according to actual treatment group, SOC and PT classification, respectively (for repeated occurrence of the same AE, the number of cases with the highest grade and the toxicity grade of the incidence will be analyzed).

d) Analysis of time to first TEAE

Time to first TEAE = date of first TEAE – date of first dose + 1. The analysis will be performed at overall level and at SOC and PT level. Only TEAEs judged as DLT will

be analyzed.

Provide detailed list of subjects with adverse events and detailed list of subjects with SAE.

3) Laboratory tests

For continuous variables, number of cases, mean, standard deviation, median, quartiles, minimum and maximum will be described at baseline and each visit by dose group, and mean, standard deviation and 95% CI will be described for changes from baseline.

4) 12-Electrocardiogram

For ECG continuous variables, number, mean, standard deviation, median, quartiles, minimum and maximum will be described at baseline and each visit by dose group, and mean, standard deviation and 95% CI will be described for changes from baseline.

The ECG examination results at each visit will be descriptively summarized for each dose group, and classified into normal, abnormal without clinical significance and abnormal with clinical significance. Cross-classification table will be used to compare with baseline ECG results.

5) Vital Signs and Weight

The results of vital signs and body weight will be summarized descriptively by visit for each dose group, including the mean, standard deviation, median, quartiles, minimum and maximum of observed values at each time point, and the mean, standard deviation and 95% CI will be described for the changes from baseline.

6) ECOG score

Results of ECOG scores at baseline and at each visit were summarized descriptively by dose, by score, and descriptively by frequency and percentage using cross-classification tables.

11.3.6 Concomitant Medication

Summarize the use of concomitant medications during the trial (including any

changes in concomitant medications during the screening period, new concomitant medications after screening and concomitant medications during the follow-up period) and the frequency of use of each drug. WHODrug Global will be used for coding, and frequency summary description will be performed based on SS population by anatomic classification (ATC first layer coding) and therapeutics (ATC second layer coding).

11.3.7 Evaluation of Anti-tumor Clinical Efficacy

The anti-tumor efficacy of TQB2858 injection will be preliminarily evaluated in this trial. Efficacy analyses were based on the Efficacy Assessment Analysis Set (EAS). Efficacy evaluation indicators mainly include: objective response rate (ORR), disease control rate (DCR), progression-free survival (PFS), overall survival (OS) and duration of response (DOR), 3-month ORR, etc.

Objective response rate (ORR): The proportion of objective response cases (PR + CR) in the total number of cases and its 95% CI will be calculated. The 95% CI for ORR was calculated based on the exact binomial method using the F-distribution.

Disease control rate (DCR): The proportion of disease control cases (PR + CR + SD) in total cases and its 95% CI will be calculated. The 95% CI for DCR was calculated based on the exact binomial method of the F-distribution.

Progression-free survival (PFS): PFS and its 95% CI were estimated using the Kaplan-Meier method, and Kaplan-Meier curves were plotted.

Overall survival (OS): OS and its 95% CI will be estimated using the Kaplan-Meier method and the Kaplan-Meier curve will be plotted.

Duration of response (DOR): Kaplan-Meier method was used to estimate DOR and its 95% CI, and Kaplan-Meier curve was plotted. Only analyzed for responders.

11.3.8 Biomarker Analysis

To explore the expression of CD3, CD8, CD86, CD20, CD163, PD-L1, TGF-β, CSF-1R and IL-1β in tumor tissues, and determine the presence of tertiary lymph node structure in tumor tissues based on the positive results in the above histological sections, so as to clarify the characteristics of the immunogenic microenvironment of TQB2858

in subjects with advanced high-grade sarcoma.

12. Original Document

In accordance with ICH E6, applicable regulations, and institutional requirements for the protection of subject personal information, each study site must maintain appropriate records related to the treatment and research related to this study. As part of a grant or participation in the study by CHIA TAI TIANQING Pharmaceutical Group, Nanjing Shunxin Pharmaceutical Co., Ltd., each site will permit the sponsor or its authorized representative and regulatory agency to inspect (or copy if legally permitted) the clinical records for quality review, audit, and evaluation of safety, study progress, and data validity.

The raw data are all information necessary for the reconstruction and evaluation of the clinical study and are original records of clinical findings, observations, or other activities. Examples of such source documents and data records include, but are not limited to, hospital records, laboratory notes, memoranda, subject diary cards, pharmacy dispensing records, recording of consultation meetings, recorded data from automated instruments, copies or transcriptions certified after verification as being accurate and complete, microfiches, photographic negatives, microfilm or disks, x-rays, and subject files and records kept at the participating pharmacy, at the laboratories, and at medico-technical departments.

Source documents were able to demonstrate the existence of the subject and substantiate the integrity of the data collected. Source documents are archived at the investigator's site.

Data transferred to the eCRF from source documents must be consistent with the source documents and any discrepancies must be explained. Depending on the trial, the investigator may need previous medical records or transfer records, or may need current medical records.

The investigator/institution will permit trial-related inspections, audits, IRB/IEC review, and regulatory inspection, providing direct access to all relevant source

data/documents. ECRFs/CRFs and all source documents, including disease records and copies of laboratory and medical test results must be available at all times for inspection by the sponsor's clinical research associates, auditors, and health authorities. The CRA and auditor may review all eCRFs/CRFs and written informed consents.

13. Quality Control and Quality Assurance

In order to ensure the quality of the trial, the clinical study plan was discussed and formulated by the sponsor and the investigator before the formal start of the trial. Conduct GCP training for relevant study personnel participating in the trial.

Each study site must manage study drug according to the protocol and SOPs, including receipt, dispensing, recovery, storage, and destruction (if applicable).

According to GCP guidelines, necessary steps should be taken during the design and implementation stage of the study to ensure that the data collected are accurate, consistent, complete and credible. All the observed results and abnormal findings in the clinical trial shall be timely verified and recorded to ensure the reliability of data. The instruments, equipment, reagents and standards used for various inspection items in the clinical trial shall have strict quality standards and ensure that they work in the normal state.

The investigator entered the information required by the protocol into the eCRF, and the monitor verified whether it was completed completely and accurately, and instructed the site staff to make necessary corrections and additions.

The drug regulatory authorities, the Ethics Committee, the monitor and/or auditor of the sponsor may systematically inspect the activities and documents related to the clinical trial to evaluate whether it is conducted in accordance with the trial protocol, SOP and relevant regulations, and whether the trial data are recorded in a timely, true, accurate and complete manner. The audit does not directly involve the personnel conducting the clinical trial.

14. Ethics

14.1 Regulations/Declaration of Helsinki

The investigator will ensure that the study is conducted in full compliance with the principles of "Declaration of Helsinki" or with the laws and regulations of the country in which the study is conducted, providing maximum protection to the subjects. The study must fully adhere to the principles of "Good Clinical Practice" in the ICH Tripartite Guideline (since January 1997) or local laws providing greater protection to the subjects.

14.2 Informed Consent

It is the responsibility of the investigator or designee of the investigator (if permitted by local regulations) to obtain written informed consent from each participating subject after adequate explanation of the aims, methods, anticipated benefits, and potential hazards of the study. For those subjects who are ineligible or unable to provide legal consent, written informed consent must be obtained from the legal guardian. If the subject and his/her legal guardian cannot read, an impartial witness must be present during the entire informed consent discussion. After the subject and guardian gave their verbal consent to participate in the study, the impartial witness signed the form to attest that the information on the informed consent was accurately explained and understood. The investigator or designee must also explain that the subject is free to refuse to participate or withdraw from the study at any time for any reason. If new safety information results in a significant change to the risk/benefit assessment, the informed consent form should be reviewed and updated as necessary. All subjects (including those already receiving treatment) should be informed of this new information and given a revised consent form to continue participation in the study.

14.3 Independent Ethics Committee/Institutional Review Board

The study protocol and any relevant materials provided to the subjects (such as subject information or study description) as well as any advertisements and compensations provided to the subjects will be submitted to the Independent Ethics Committee (IEC) by the investigator. Ethical approval must be obtained before the start of the study and documented in the form of a letter from the investigator, including the

date the Ethics Committee met and granted consent.

After receiving the approval letter from the Independent Ethics Committee, the investigator should also submit the protocol to the Ethics Committee according to the procedures and regulatory requirements.

15. Publication and patent of trial results

The trial results are owned by Chia Tai Tianqing Pharmaceutical Group Nanjing Shunxin Pharmaceutical Co., Ltd. The investigator (study site) should obtain the consent of Chia Tai Tianqing Pharmaceutical Group Nanjing Shunxin Pharmaceutical Co., Ltd. before publishing the paper. Before the main study results are officially published, the investigator (study institution) should exchange the clinical trial results at the academic conference with the prior consent of Chia Tai Tianqing Pharmaceutical Group Nanjing Shunxin Pharmaceutical Co., Ltd.

The patent application for the technical solution (including but not limited to the selection of indications and dose) agreed in this project shall be exclusively owned by CHIA TAI TIANQING Pharmaceutical Group CO., LTD. In the process of clinical trials, clinical research institutions shall jointly apply for patents with CHIA TAI TIANQING Pharmaceutical Group CO., LTD for innovative achievements independently completed by the agreed clinical protocol. CHIA TAI TIANQING Pharmaceutical Group CO., LTD and its Affiliates may manufacture, use, sell, sell, commercialize the products covered by the above patents or those obtained by patent methods. However, without the permission of either party, neither party may transfer or in any way permit a third party to exploit such patent.

16. Estimated Trial Schedule

Proposed start time: November 2021 (after approval by the Ethics Committee) .

Estimated duration of trial: November 2021-November 2023.

References

1 Lu D, Ni Z, Liu X, Feng S, Dong X, Shi X *et al*. Beyond T Cells: Understanding the Role of PD-1/PD-L1 in Tumor-Associated Macrophages. *J Immunol Res* 2019; 2019: 1919082.

2 Gandini S, Massi D, Mandalà M. PD-L1 expression in cancer patients receiving anti PD-1/PD-L1 antibodies: A systematic review and meta-analysis. *Crit Rev Oncol Hematol* 2016; 100: 88-98.

3 Liu S, Chen S, Zeng J. TGFbeta signaling: A complex role in tumorigenesis (Review). *Mol Med Rep* 2018; 17: 699-704.

4 Tawbi H A, Burgess M, Bolejack V, et al. Pembrolizumab in advanced soft-tissue sarcoma and bone sarcoma (SARC028): a multicentre, two-cohort, single-arm, open-label, phase 2 trial. Lancet Oncology, 2017, 18 (11): 1493-1501.

Attachments One calculation formula

1. Cockcroft-Gault creatinine clearance calculation formula

Ccr = [(140-age) × body weight (kg)]/[72 × Scr (mg/dL)], unit: mL/min

Note: (calculated result × 0.85 for female subjects)

2. QTc calculation Bazetts correction formula

QTc = QT/(RR0.5); RR is normalized heart rate value, RR = 60/heart rate

APPENDIX II: EVALUATION OF QUALITY OF LIFE (ECOG PS) (5 POINTS)

0	Normal activity
1	Mild symptoms, self-care, able to engage in light physical activity
2	Can tolerate the symptoms of the tumor and take care of himself/herself, but can stay in bed for no more than 50% of the time during the day
3	The tumor symptoms are severe, with more than 50% of the time in bed during the day, but they can also get up and stand, and some of them take care of themselves
4	Critically ill Bedridden
5	Death

APPENDIX III NEW YORK HEART ASSOCIATION (NYHA) FUNCTIONAL CLASSIFICATION

Grading	New York Heart Association (NYHA) Class
Grade I	No limitation of physical activity, ordinary activity does not cause undue fatigue, dyspnea or palpitation. That is, the compensatory phase of cardiac function.
Grade II	Slight limitation of physical activity. Asymptomatic at rest, fatigue, palpitation, dyspnea, or angina may be precipitated by ordinary activity. It is also known as grade I or mild heart failure.
Grade III	Marked limitation of physical activity, absence of symptoms at rest, less than ordinary activity producing the above symptoms. Also known as grade II or moderate heart failure.
Grade IV	Inability to engage in any physical activity, congestive heart failure or angina symptoms at rest, aggravated by any physical activity. It is also known as grade III or severe heart failure.

Note: Cardiac function is divided into four classes, and heart failure is divided into three classes (slightly supplemented according to NYHA classification)).
